# Trustworthy Multimodal Attention Framework for Creep Rupture Life Prediction Under Data‐Scarce Conditions: A Case Study on IN718

**DOI:** 10.1002/advs.77026

**Published:** 2026-08-10

**Authors:** Haopeng Lv, Dayong Wu, Ziyuan Rao, Qian Wang, Haikun Ma, Jie Kang, Wang Li, Huicong Dong, Yandong Wang, Zhinan Yang, Ru Su

**Affiliations:** ^1^ Hebei Short Process Steelmaking Technology Innovation Center School of Materials Science and Engineering Hebei University of Science and Technology Shijiazhuang People's Republic of China; ^2^ National Engineering Research Center of Light Alloy Net Forming Shanghai Jiao Tong University Shanghai People's Republic of China; ^3^ State Key Laboratory for Advanced Metals and Materials University of Science and Technology Beijing Beijing People's Republic of China; ^4^ Hebei Lron and Steel Laboratory North China University of Science and Technology Tangshan China; ^5^ Yanzhao Lron and Steel Laboratory Tangshan People's Republic of China

**Keywords:** attention mechanism, creep life prediction, interpretability, materials design, multimodal deep learning

## Abstract

An attention‐based multimodal deep learning framework is developed to fuse processing parameters with microstructural micrographs for predicting creep rupture life of IN718 under a fixed creep testing condition. Under a predefined composition‐stratified, sample‐level split, the framework achieved a mean test‐set *R^2^* of 0.917 ± 0.014 across 50 random‐seed training repetitions. The corresponding mean *RMSE* and *MAPE* were 0.14% and 6.0%, respectively. Interpretability analyses suggest that the predictions are consistent with established metallurgical understanding, particularly the important role of δ‐phase characteristics. Furthermore, uncertainty quantification endows the model with self‐assessment capabilities, allowing it to reliably quantify the confidence of its predictions. This study establishes a methodological framework that unifies predictive accuracy, physical interpretability, and model confidence, providing a validated paradigm for developing trustworthy AI models for materials design under data‐limited conditions.

## Graphical abstract

An attention‐based multimodal deep learning framework is developed to predict the creep life of Ni‐based superalloys by fusing processing parameters with microstructural micrographs. The model achieves high accuracy (R^2^ = 0.92), aligns with metallurgical principles by capturing δ‐phase influence, and incorporates uncertainty quantification, offering a trustworthy AI paradigm for data‐limited materials design.

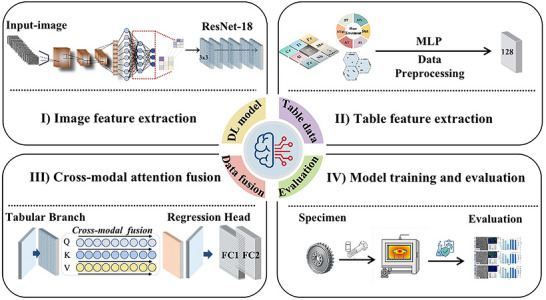

## 1 Introduction

The ability to accurately predict material properties is fundamental to the design and deployment of high‐performance components in advanced engineering systems [1, 2]. However, this task remains a long‐standing challenge, as macroscopic performance is governed by a complex, nonlinear interplay between composition, processing history, and the resultant microstructure [3, 4]. While data‐driven machine learning (ML) offers a powerful paradigm for navigating this complexity, its current application is often constrained by reliance on single‐modality data [5, 6, 7]. For metallic materials, models that rely on tabulated processing and composition data inherently overlook essential morphological information encoded in microstructural micrographs. Conversely, image‐based models, while adept at capturing structural detail, decouple this information from the crucial processing context in which it was created [8, 9, 10]. This fundamental modality disconnect obstructs a holistic understanding of material behavior and intrinsically limits both predictive accuracy and generalization potential of existing models [11, 12].

Attempts to mitigate the limitations of single‐modality data have been explored, yet with limited incremental success. For instance, physics‐informed learning and data augmentation techniques have been employed to enhance model performance in data‐scarce scenarios [13, 14, 15]. However, these strategies primarily operate within a single data type and do not address the core challenge of integrating disparate information sources. An alternative pathway, high‐throughput experimentation, aims to circumvent the small data problem by generating vast datasets [16, 17, 18, 19, 20]. Nevertheless, its prohibitive costs and protracted experimental cycles render it impractical for widespread application to complex alloy systems where sample fabrication and property characterization are resource‐intensive [21]. Consequently, a critical gap persists: the lack of a framework capable of synergistically fusing quantitative, tabulated processing data with high‐dimensional, qualitative microstructural micrographs, especially under the typical data‐limited conditions of materials research.

The recent advent of multimodal deep learning offers a powerful paradigm to bridge this gap [22, 23]. By designing architectures capable of synergistically integrating heterogeneous data, this methodology holds the potential to construct a more holistic representation of a material system, thereby unlocking a deeper understanding of the process‐structure‐property relationship [24]. While this approach has achieved considerable success in classification tasks within other scientific domains, such as biomedicine [25], its application to complex, regression‐based property prediction in materials science remains nascent. To evaluate the potential of this emerging paradigm, the present study selects a quintessential and challenging problem as a test case: predicting creep rupture life in the Ni‐based superalloy IN718. This system serves as an ideal exemplar because its high‐temperature mechanical performance is highly sensitive to subtle variations in both processing parameters and the resultant δ‐phase morphology, making it a prime candidate for validating the efficacy of a multimodal fusion approach [26].

To this end, a multimodal framework centered on a multimodal attention mechanism is developed and validated. The architecture is specifically designed to model the dependencies between processing parameters and microstructural features explicitly. This design goes beyond simple feature concatenation by allowing the tabular/process embedding to weight spatial image tokens through multimodal attention. Applied to the IN718 creep rupture life prediction task, the framework demonstrates strong predictive accuracy, achieving a mean absolute relative error of less than 6% on an unseen test set. More significantly, the interpretability of the model is enhanced through a dual, synergistic interpretability analysis. Gradient‐weighted Class Activation Mapping (Grad‐CAM) [27] visualizations reveal discriminative image regions in the final convolutional feature maps that contribute to the prediction. Concurrently, a descriptor‐based post hoc Shapley Additive Explanations (SHAP) [27] analysis performed on a Random Forest model quantitatively associates the MDLF predictions with physically meaningful microstructural descriptors. The resulting interpretation is consistent with established metallurgical understanding: the model predictions show to the non‐linear influence of δ‐phase characteristics, including the beneficial effect of suitable grain‐boundary pinning morphologies and the reduced strengthening effect associated with δ‐phase dissolution at high solution‐treatment temperatures. Therefore, this work establishes a proof‐of‐concept, contributing a validated methodological blueprint for building not only accurate but also physically‐informed and trustworthy predictive models in materials science, particularly within the challenging context of data‐limited research.

**FIGURE 1:**
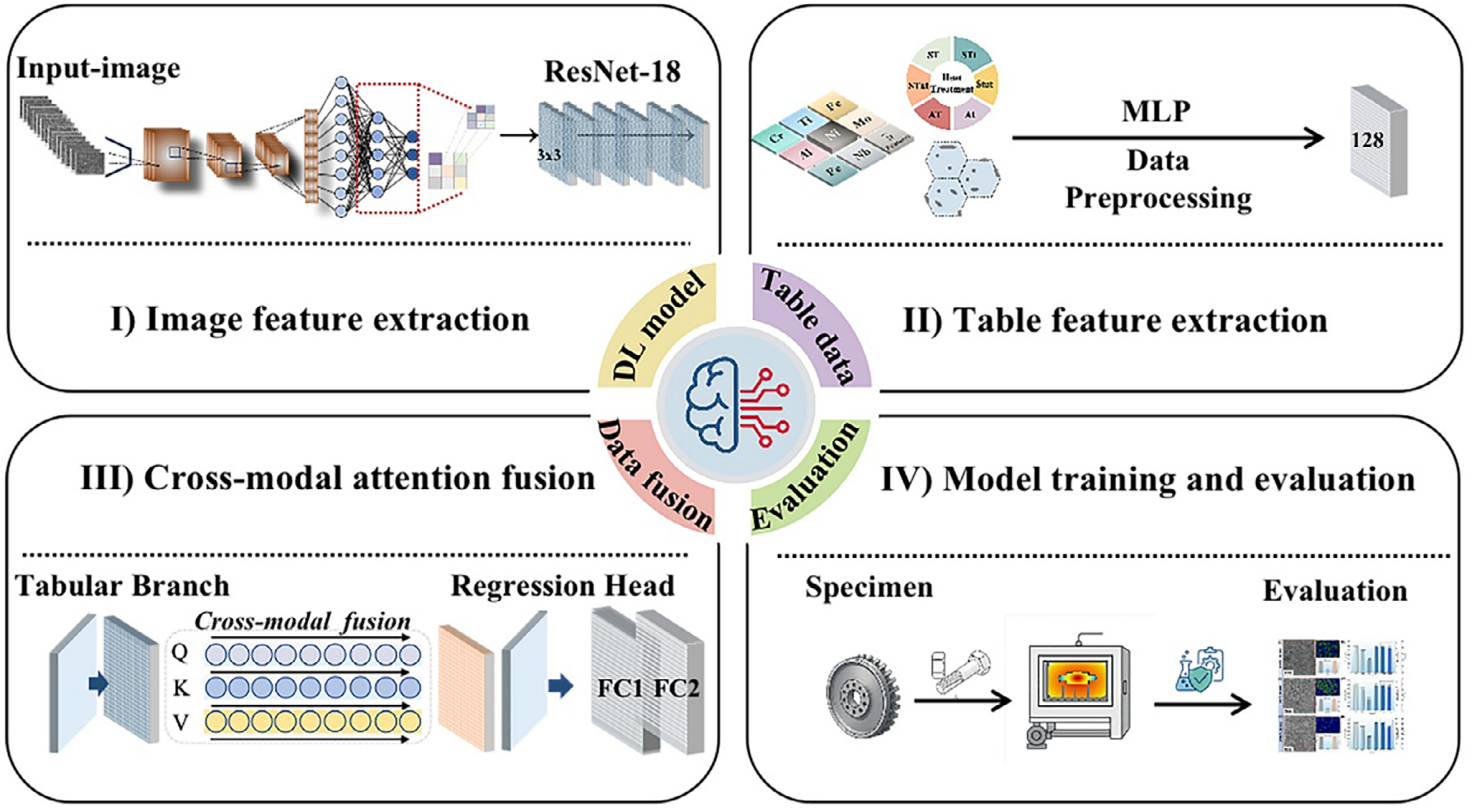
Architecture of the proposed MDLF. The normalized composition and heat‐treatment variables are encoded into a 128D tabular embedding that serves as a single Query token. The 7 × 7 Stage‐4 image feature map is flattened into 49 spatial tokens that serve as the Keys and Values after linear projection. Multimodal attention produces a 128D tabular‐conditioned representation, which is concatenated with the 512D globally pooled image representation to form the 640D input to the regression head. I) and a tabular data stream, embedded by an MLP II). These streams are intelligently fused in the multimodal attention core III), where processing information guides the focus of the model on salient visual features. The entire framework is trained and validated against physical experiments IV) to predict the creep rupture life of the IN718 superalloy.

As illustrated in Figure 1, the Multimodal Deep Learning Framework (MDLF) is organized into four key modules: I) an image feature extraction backbone; II) a tabular data embedding branch; III) a multimodal attention fusion core, and IV) a final regression head for prediction.

1) Given the data‐limited context of this study, a transfer learning approach was adopted to leverage knowledge from a large‐scale dataset and mitigate overfitting [28]. A ResNet‐18 model was employed, pretrained on ImageNet [29, 30, 31], as the image feature extraction backbone (details in Section S1). To adapt this backbone for metallurgical image analysis, a tailored fine‐tuning strategy was implemented. The shallow convolutional layers (Stages 1–2), which extract general, low‐level features such as edges and textures, were initially frozen. In contrast, the deeper layers (Stages 3–4) were trained to learn domain‐specific, high‐level features pertinent to δ‐phase morphology and distribution. After passing through the network layers, the output from the final convolutional block was extracted, encoding each image tile into a spatially‐preserved 512‐channel feature map.

2) Projection of normalized tabular data into a dense, 128D embedding space was accomplished through a two‐layer multi‐layer perceptron (MLP) configured with batch normalization and ReLU activation [32]. The dimensionality balances feature resolution with computational cost. This design suits the requirements of the downstream attention blocks. Additionally, a dropout rate of 0.2 was applied to the embedding layer. This step reduces bias toward the specific modality during early training epochs. As a result, the network establishes more general multimodal dependencies.

The multimodal attention module performs tabular‐conditioned aggregation of spatial image features. The normalized composition and heat‐treatment variables are encoded into a 128D sample‐level embedding, which serves as a single Query token. The 7 × 7 Stage‐4 feature map from ResNet‐18 is flattened into 49 spatial image tokens. These tokens are linearly projected to form the Keys and Values in the same 128D attention space. Scaled dot‐product scores are normalized over the 49 image tokens and the resulting weights determine their contributions to the tabular‐conditioned image representation.

Eight attention heads were used. Their outputs were concatenated, projected to 128 dimensions and combined with the original tabular embedding through residual addition and layer normalization. In parallel, global average pooling of the Stage‐4 feature map produced a 512D image‐level representation. Concatenation of the 512D image representation and the 128D fused representation yielded the 640D input to the regression head. The complete tensor dimensions and equations are provided in Section S2.

4) The framework was trained end‐to‐end using the AdamW optimizer (initial learning rate 1 × 10−3, weight decay 1 × 10−4) and a ReduceLROnPlateau learning rate scheduler. To mitigate overfitting, differentiated dropout rates (0.2 for tabular embedding, 0.3 for the final regression layers) and an early stopping policy (patience of 40 epochs) were employed.

The primary evaluation used a predefined composition‐stratified, sample‐level split of 94 training, 20 validation, and 20 test samples. All data derived from the same sample were retained within one subset. The partition remained fixed across 50 random‐seed training repetitions. The tabular‐only, image‐only, concatenation‐based multimodal, and MDLF models were evaluated using the same sample partition. Five‐fold cross‐validation and a stratified generalization protocol were conducted separately as supplementary analyses.

For physical interpretation, a Random Forest model was trained using post hoc quantitative microstructural descriptors as inputs and the corresponding MDLF predictions as target outputs. SHAP analysis was performed on the model.

## 2 Results

### 2.1 Creep Rupture Life Prediction

Under this fixed partition, the model was trained 50 times using different random seeds. Across these repetitions, the mean test‐set *R^2^* was 0.917 ± 0.014, with a 95% confidence interval of (0.913, 0.921) (See Figure S3 in Section S3), while the mean test‐set *RMSE* and *MAPE* were 0.14 and 6.0%, respectively. The mean *R^2^* was rounded to the headline value of 0.92. Because the sample partition remained unchanged, the variation across runs reflects stochastic training variability rather than variation in data partitioning.

**FIGURE 2:**
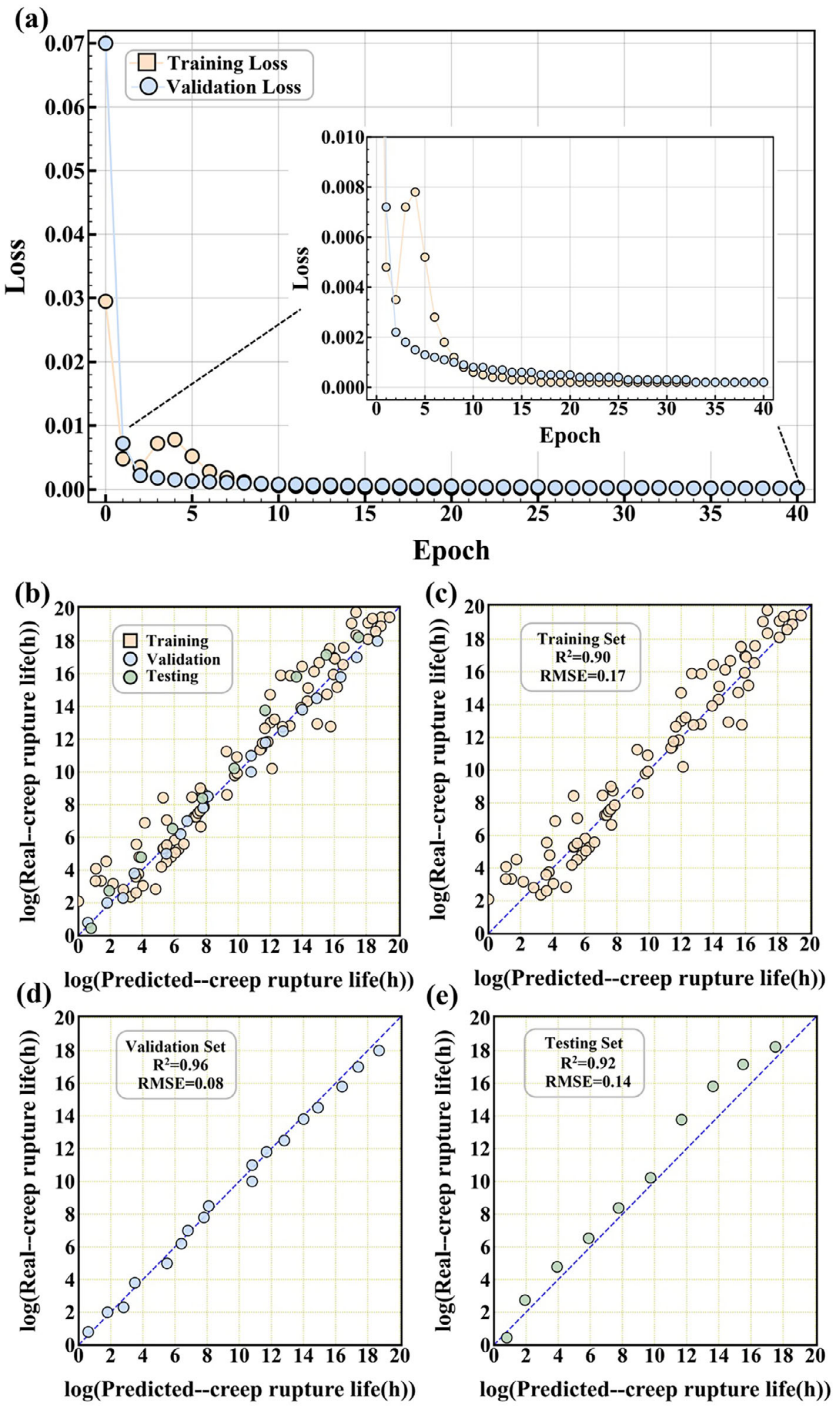
Performance of the MDLF under the predefined composition‐stratified, sample‐level split. The dataset was divided into 94 training, 20 validation, and 20 test samples, and the partition remained fixed across 50 random‐seed training repetitions. (a) Training and validation losses from a representative run. (b–e) Parity plots for the combined, training, validation, and test subsets of the same run.

Figure 2a shows the training and validation losses of a representative run and Figure 2b–e show the corresponding parity plots. Five‐fold cross‐validation was conducted separately and yielded fold‐averaged values of *R^2^* = 0.92 and *RMSE* = 0.18. These results are reported in Section S4 and are not the source of the headline performance.

### 2.2 Model Validation

**FIGURE 3:**
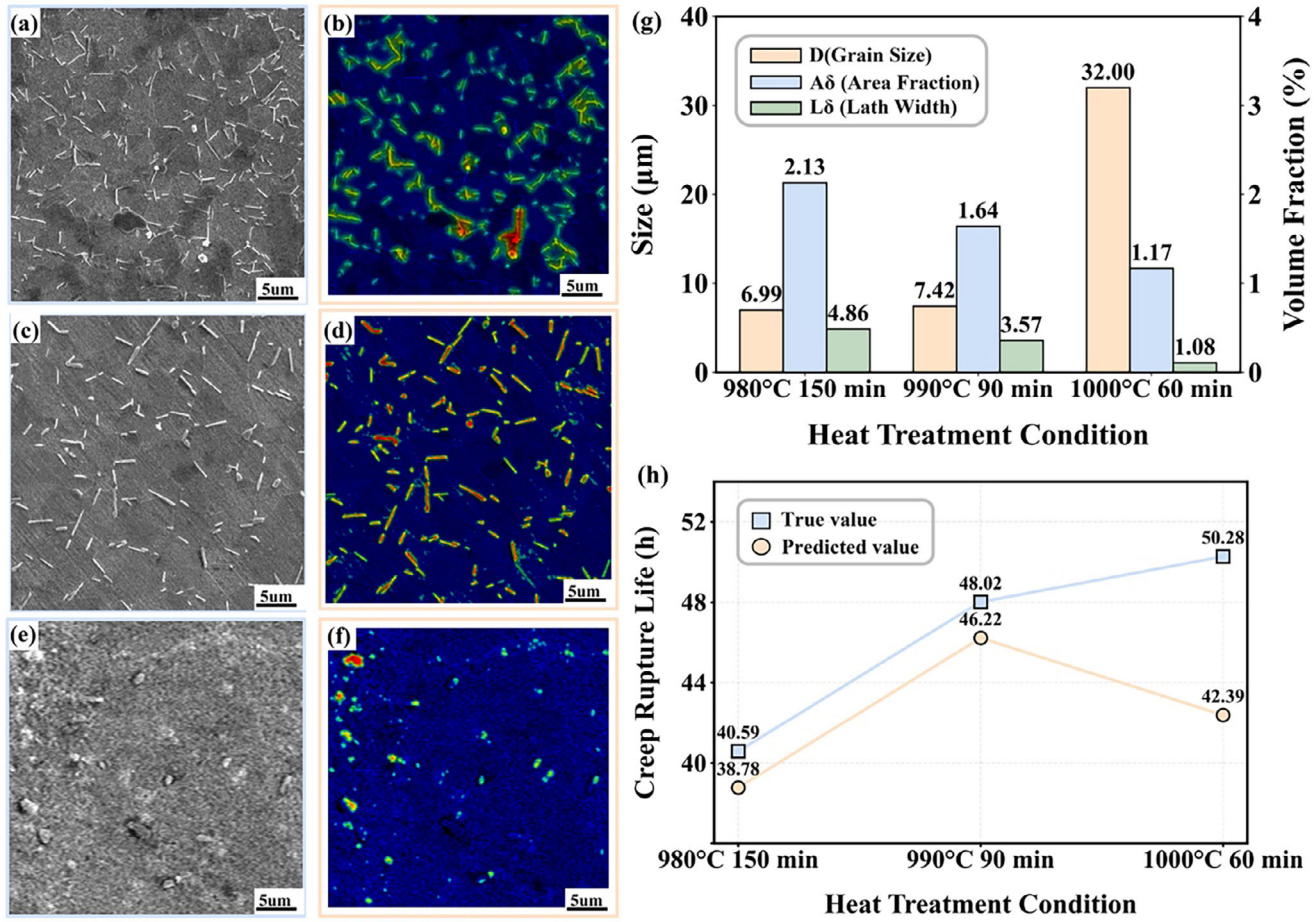
Influence of different solution heat treatments on the microstructure and creep properties of IN718 alloy and the prediction results of the model. (a, c, and e) are the SEM micrographs after treatments at 980°C for 150 min, 990°C for 90 min and 1000°C for 60 min, respectively. (b, d, and f) are the corresponding heat maps of microstructural features, regions with high Grad‐CAM saliency. (g) presents a quantitative comparison of microstructural parameters under the different treatment conditions, including D, A*_δ_*, and L*_δ_*. (h) displays a comparison between the experimental creep rupture life (True value) and the values predicted by the model (Predicted value).

To rigorously assess the generalization capability of the MDLF, the framework was evaluated on a series of IN718 samples synthesized under processing conditions excluded from the training database (Figure 3). Predictions remain accurate when the δ‐phase retains morphological stability. For samples treated at 980°C (150 min) and 990°C (90 min), relative errors were low at 4.4% and 3.3%, respectively. However, results for the 1000°C (60 min) condition deviated significantly: the relative error increased to 15% (predicted: 42.39 h; actual: 50.28 h). This decline indicates the generalization limit of the model. Severe microstructural evolution likely explains this outcome‐partial dissolution of the δ‐phase shifts the feature space outside the distribution covered by the training dataset.

Predict errors at 1000°C expose the limitations of the standard deterministic regression model. Such models cannot quantify uncertainty when applied to new data. To address this issue, this study introduced a method of quantifying uncertainty (UQ) [33] and used Monte Carlo (MC) Dropout technology [34] to give the MDLF framework the capability of quantifying uncertainty, as detailed in Section S5. The core idea of this technology is to maintain the randomly activated state of the Dropout layer during the model reasoning phase. Figure S6 shows that the prediction results of 980°C and 990°C samples show a small standard deviation after MC Dropout technology is applied, indicating a high confidence level of the model; In contrast, the predicted standard deviation for the 1000°C sample is significantly increased, which accurately reflects the low confidence of the model in the predictions outside this distribution.

SEM micrographs (Figure 3a,c,e) reveal significant microstructural evolution at 1000°C. This phenomenon leads to deviations in performance predictions. In contrast, microstructures at 980°C and 990°C retain distinct δ‐phase precipitates. These features provided informative visual cues for the image branch of the MDLF. On the other hand, heating to 1000°C approached the δ‐phase solvus temperature and led to the near‐total dissolution of this phase. The resulting homogenized structure reduced the amount of discriminative microstructural information available to the image branch. Consequently, the predictions relied heavily on numerical processing data. However, this input format proved insufficient for resolving the non‐linear structure‐property relationships in this regime. These results confirm the generalization ability of the MDLF framework within the calibration range but highlight the requirement for comprehensive data from both modalities.

### 2.3 Mechanistic Insights via Microstructure Visualization

Although MDLF models show excellent prediction accuracy, the inherent complexity of deep neural networks often makes them seen as “black boxes.” The high‐dimensional features learned by the models are often difficult to represent, making it difficult for researchers to understand the underlying decision mechanisms associated with a particular microstructure with predicted performance.

**FIGURE 4:**
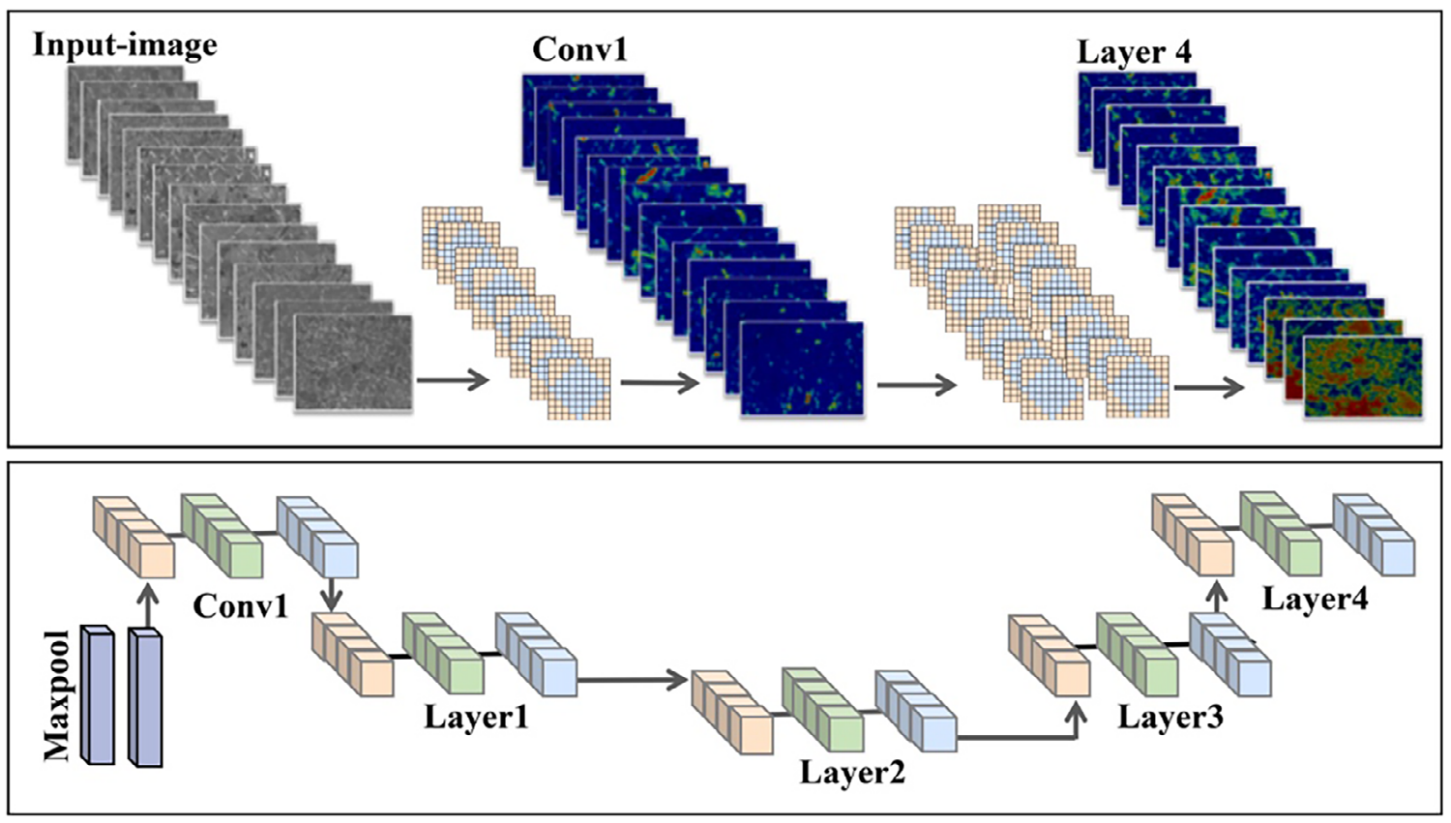
Schematic representation of the ResNet‐18 architecture and feature map visualization. The top panel depicts the evolution of input micrographs into feature maps at various network depths, including the Conv1 layer and Layer 4. The bottom panel displays the configuration of the ResNet‐18 module. Data passes through sequential convolutional layers and residual connections. This process generates the feature maps.

To resolve this, Grad‐CAM techniques were utilized. These techniques visualize key regions in the input microstructure. The visualization reveals the parts critical to the prediction process. As described in the Method Section, this technique generates a “significance map” or “thermal map” (Figure 4) corresponding to the original input image. In this thermogram, the high‐intensity region (e.g., the red or yellow‐marked part) has a significant impact on the microstructure features that contribute to the final prediction, while the low‐intensity region (e.g., the blue‐marked part) represents the less contributing features. Superimposed on the original SEM micrographs, these thermograms allow us to explore discriminative image regions identified by Grad‐CAM and assess whether the feature extraction mechanism is consistent with established metallurgical principles. Detailed analysis based on this visualization method is presented below.

#### 2.3.1 Identification of Key Phase

**FIGURE 5:**
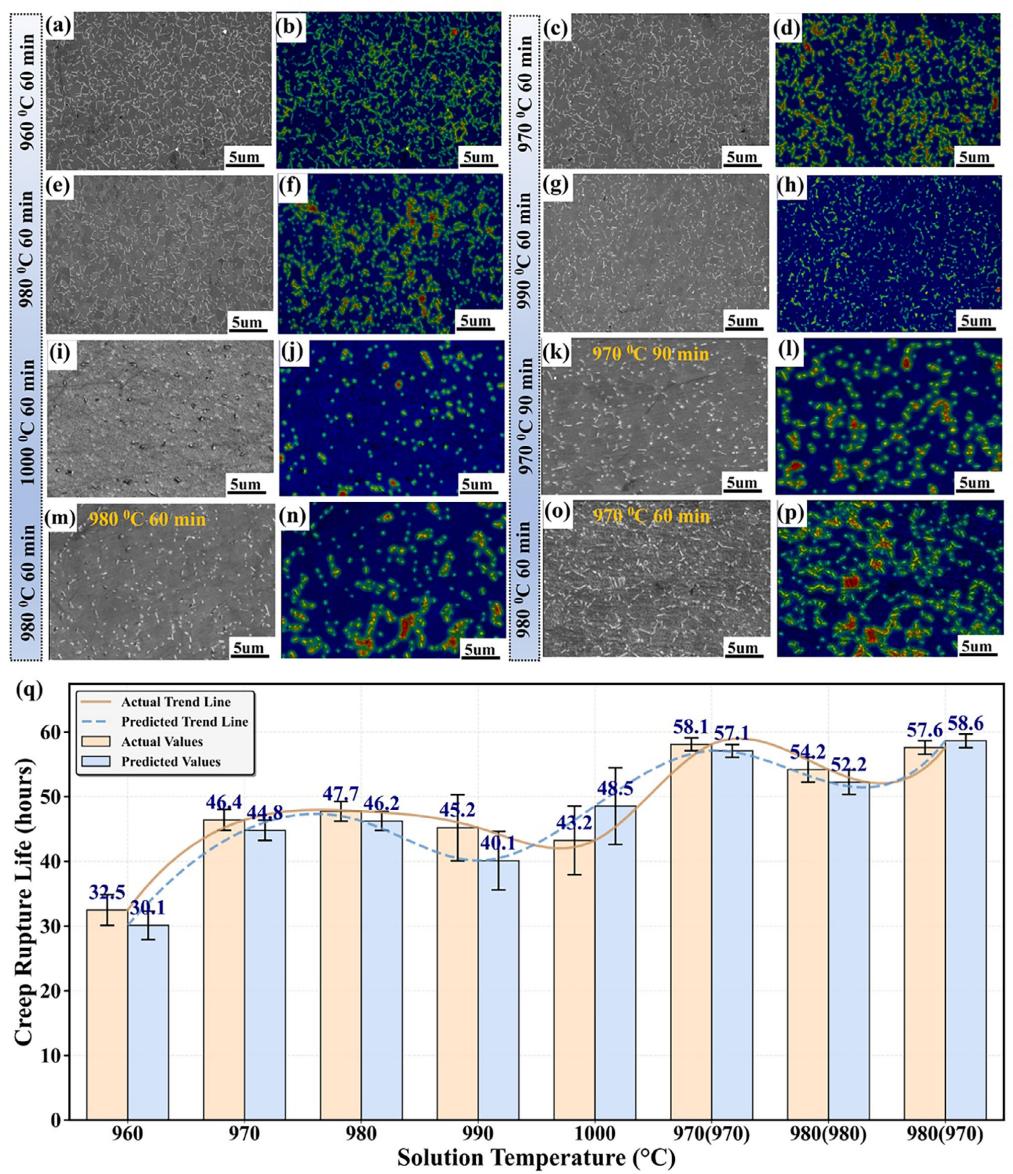
Grad‐CAM visualization of the final convolutional feature maps. (a–p) Paired SEM micrographs and corresponding Grad‐CAM saliency maps of IN718 alloys after different solution treatments. Blue and red regions indicate low and high Grad‐CAM saliency, respectively. High‐saliency regions are mainly located near δ‐phase‐related microstructural features. (q) Comparison between experimental and predicted creep rupture life under different treatment conditions, showing that the model follows the observed non‐linear trend.

The interpretability of the MDLF model was evaluated by visualizing its inference process using Grad‐CAM. The results indicate that the feature representations captured by the model align with physical metallurgical principles, distinguishing it from models that rely on spurious background correlations. Figure 5 shows that many high‐activation regions overlap with precipitate‐rich regions. The high‐activity areas in the heat maps (yellow to red) line up with the δ‐phase particles. Importantly, the hot spots are not confined to the particles but extend into the immediate surrounding matrix, indicating that the architecture captures a key physical concept. A standard segmentation tool would outline the shape of a particle. Conversely, the model learned that the strengthening from the δ‐phase is non‐local. This effect is created by how the phase interacts with the surrounding matrix through strain fields and pinning. The presence of high Grad‐CAM saliency near these interaction zones suggests that the image branch uses microstructural features relevant to creep rupture life in IN718.

The Grad‐CAM saliency patterns vary with processing history and the associated microstructural evolution, rather than remaining identical across treatment conditions. Specifically, increasing the temperature toward the δ‐phase solvus reduces both the area and intensity of high‐activation regions (Figure 5e–j). This reduction parallels the physical dissolution of the δ‐precipitates. Such alignment indicates that the model captures the function of the δ‐phase as a strengthening agent rather than simply extracting morphological shapes. Consequently, the framework accurately reproduces the non‐linear creep rupture life trends observed experimentally (Figure 5q). The findings reflect precipitation strengthening effects. Additionally, the results account for property degradation caused by phase dissolution at high temperatures.

It should be noted that Grad‐CAM and the multimodal attention mechanism provide different forms of model interpretation. Grad‐CAM highlights discriminative image regions derived from the gradients of the final convolutional feature maps, whereas the learned multimodal attention weights describe the allocation of attention between the tabular processing information and the spatial image tokens. To further distinguish these two quantities, the actual multimodal attention weights are visualized separately in Section S6.

#### 2.3.2 Differentiating Complex Morphologies

Further tests on the model used a two‐stage solution treatment, which produced materials with complex heterogeneous microstructures. Fine and scattered δ‐phases were generated at 970°C (Figure 5c,d), while larger and mid‐sized precipitates formed at 980°C (Figure 5e,f). The resulting Grad‐CAM maps (Figure 5k–p) show the capability of the framework to process this complexity: the strongest Grad‐CAM activations are assigned to mid‐sized δ‐phases, appearing as bright red spots on the maps, which are known to be more effective for pinning grain boundaries and lower importance to smaller particles that manifest as scattered yellow spots. The model can distinguish the influence of different particle morphology and size. These results suggest that the image features used by the model are consistent with physical metallurgical understanding. Grad‐CAM therefore provides a useful qualitative visualization of how image regions are associated with the model prediction.

#### 2.3.3 Capturing Non‐Linear Temporal Evolution

**FIGURE 6:**
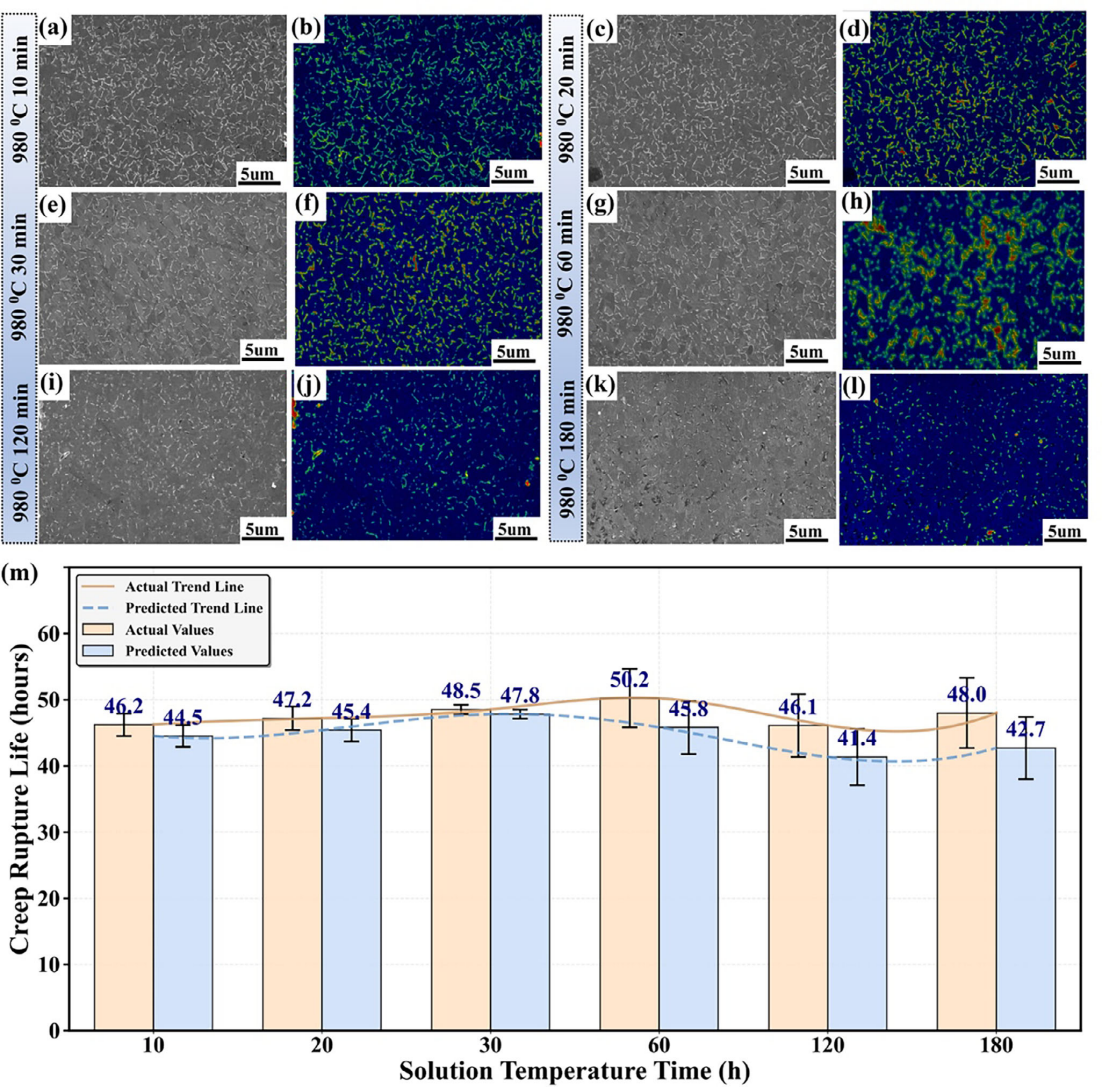
This figure shows the microstructure corresponding to different holding times at 980°C. (a–l) presenting paired SEM micrographs and their corresponding Grad‐CAM thermograms; (m) The experimental creep rupture life is compared with the predicted creep rupture life, which reflects the ability of the model to capture nonlinear time trends.

To complement the temperature‐dependent analysis, we checked how the model handles changes over time by using a fixed temperature of 980°C but varying the solution treatment time. As shown in Figure 6, the results reveal that the model effectively captures the trends of microstructural change well. In the first 10–30 min, the microstructure changes, starting with many fine, needle‐shaped δ‐phases and ending with coarser, block‐shaped structures (Figure 6a,c,e). The Grad‐CAM heat maps correctly follow this change (Figure 6b,d,f). The hot spots on the map start out scattered over the fine particles and then become more focused and intense on the coarser δ‐phases. This indicates that the model has established correlations between different particle shapes with their different effects on creep resistance.

The creep rupture life reaches its peak at a 60 min holding time (Figure 6m). At this point, the δ‐phase has formed coarse, blocky structures, which is the most effective for pinning grain boundaries (Figure 6g). The matching heat map (Figure 6h) shows a very focused and intense hot spot, suggesting the model correctly found that this shape is the key to achieving the best creep life. This shows the model learned to link this specific δ‐phase structure to the peak performance of the material. When the treatment time extends beyond than 60 min (to 120–180 min), much of the δ‐phase dissolves, causing a large drop in both its amount and size (Figure 6i,k). As demonstrated in previous studies [35, 36, 37], this process weakens the pinning effect at the grain boundaries, making the material more likely to fail from creep at high temperatures and thus shortening its rupture life. The dynamic Grad‐CAM activation patterns of the model closely mirrors the microstructural evolution observed in the material, as reflected in the Grad‐CAM micrographs (Figure 6j,l): the activation intensity of the hot spots decreases and they become scattered again, mirroring the fact that the strengthening phase is disappearing. This reveals that the model is not limited to simply following data trends, but incorporates deeper physical mechanisms. Specifically, the model extracted the strong non‐linear correlation where a decrease in the size and volume fraction of the δ‐phase fall below a specific critical value, its strengthening effect on the matrix will significantly decrease.

The experimental data strongly confirms the direct correspondence between the dynamic Grad‐CAM activation patterns distribution within the model and the classical physical metallurgical principles governing the IN718 alloy. Essentially, the core function of this model is to identify and quantitatively characterize key features closely related to the physical mechanisms that dominate material properties. This multimodal computing framework effectively overcomes the limitations of traditional digital image processing techniques, which are typically limited to recognizing morphological and geometric features of the δ‐phase. In contrast, this framework can achieve deep encoding of multidimensional functional saliency features in a panoramic microstructural background spanning macro to micro scales. This ability is demonstrated in the precise characterization of the nonlinear strengthening effect in high‐temperature creep resistance.

This is substantiated by how the model tracks the entire lifecycle of the effectiveness of the δ‐phase. The Grad‐CAM micrographs in Figure 6 show the coarsening process. The Grad‐CAM saliency becomes more concentrated as the δ‐phase particles evolve from scattered needle‐like precipitates into coarser blocky morphologies associated with higher creep resistance. The micrographs in Figure 5 show the point where the phase starts to dissolve. As the temperature increases and δ‐phase dissolution becomes more pronounced, the Grad‐CAM saliency associated with δ‐phase‐rich regions decreases. This reflects the physical loss of the phase that strengthens the material. Across these processes, the repeated occurrence of high Grad‐CAM saliency near particle‐matrix and grain‐boundary regions suggests that these regions contribute to the model prediction.

This validation confirms that deep learning models can extract and synthesize multi‐scale, dynamic microstructural information in a complex alloy system. It provides quantitative evidence for the non‐linear correlations between the entire process‐structure evolution and macroscopic performance, thereby providing a reliable and physically grounded theoretical foundation for ML‐driven materials design.

## 3 Discussion

The performance and underlying mechanisms of the MDLF are discussed herein. The analysis begins by establishing the superiority of the multimodal approach through a series of comparative benchmarks, followed by an interpretability‐driven investigation into the model internal reasoning process.

**FIGURE 7:**
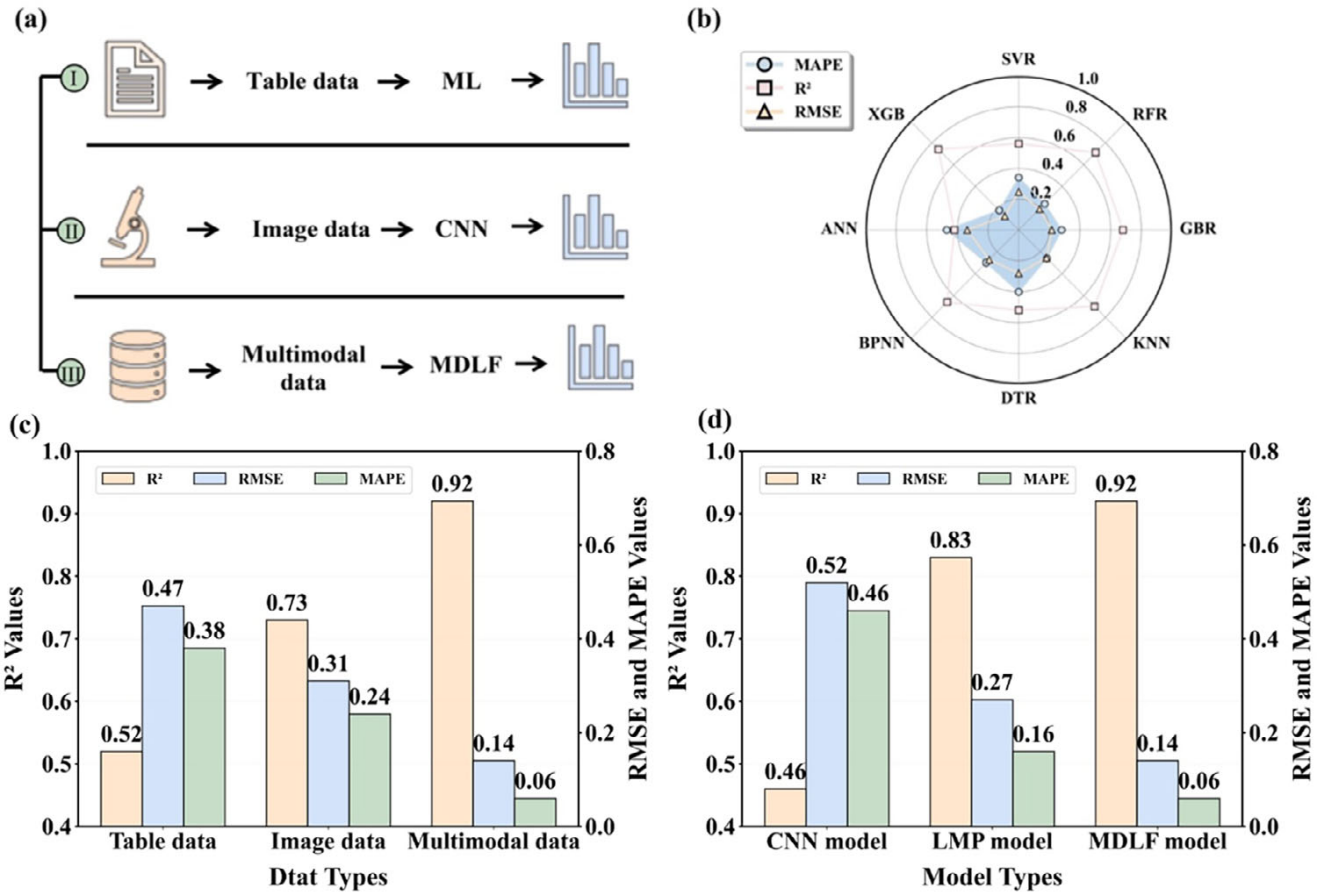
Comparison of the MDLF with data‐driven baselines and an external empirical reference. (a) Schematic comparison of the modeling approaches. (b) Performance of the tabular data‐driven models. (c) Same‐split comparison of the tabular‐only, image‐only, concatenation‐based multimodal, and MDLF models. The models in (b,c) were evaluated using the same predefined sample partition. (d) Larson‐Miller Parameter model calibrated using a separate multi‐condition database and presented as an external empirical reference.

Comparative benchmark analyses were performed to evaluate the MDLF (Figure 7). These tests included ablation studies and comparisons with traditional machine learning and physics‐based models. The effect of data fusion is shown in Figure 7c. A model trained solely on tabular data (composition and processing parameters) yielded a low average *R* ^2^ of 0.52 using standard ML algorithms. To rule out model capacity limitations, we further evaluated the tabular foundation model (TablePFN). While effective in interpolation tasks, its performance dropped significantly under the rigorous stratified generalization protocol employed in this study (detailed analysis in Section S7 and Figure S8), confirming that tabular features alone cannot capture the stochastic variability of microstructural evolution. The processing history contains information, but the predictive accuracy is limited by the absence of microstructural descriptors. The model trained on image data resulted in a mean *R* ^2^ of 0.73. Furthermore, to assess training stability and possible overfitting, we evaluated the performance gap between the training, validation, and test sets across 50 independent random‐seed repetitions under the predefined sample‐level split for the stochastic baseline models (see Section S8 and Figure S9). The statistical results clearly reveal that relying solely on a single modality (e.g., TablePFN and pure vision models) leads to pronounced overfitting with significant generalization gaps on this limited dataset. In stark contrast, our MDLF mitigates this bottleneck through multimodal regularization. The microstructure contains features related to performance, but the accuracy is restricted by the lack of processing parameters. The multimodal fusion model resulted in an average *R* ^2^ of 0.92. This is an increase of 0.40 and 0.19 compared to the tabular and image models. The *RMSE* was reduced to 0.14 and the MAPE to 6%. These results indicate that the integration of process and structure data is necessary for the modeling of material properties.

We conducted framework validation by benchmarking against the XGBoost regressor (XGR), which is a typical traditional algorithm limited to table inputs and achieved a significantly lower mean *R* ^2^ value of 0.80 (Figure 7b). However, these methods require manual feature engineering. An expert must choose the relevant features ahead of time. This approach makes it difficult for the model to find hidden, complex patterns in the material data. The direct extraction of features from raw data by MDLF reduces reliance on manual engineering. This process makes a stronger, more direct connection between the microstructure and the final performance.

To provide a physics‐based empirical reference, the MDLF was compared with a Larson‐Miller Parameter (LMP) baseline, which is commonly used to describe creep rupture behavior through a stress‐temperature‐time relationship. The LMP model relates rupture time, temperature, and applied stress through a time‐temperature parameter and provides a physics‐based empirical baseline for creep rupture prediction.

Although the proposed MDLF and the LMP model are established using different modeling paradigms, both approaches were evaluated on the same target prediction task under the identical fixed creep‐testing condition (650°C/690 MPa). Therefore, the comparison presented in Figure 7d reflects differences in predictive capability under the same evaluation protocol. Additional details regarding the construction and evaluation procedure of the LMP baseline are provided in Section S8. The model showed robust performance on the test set, with an average *R* ^2^ values of 0.83 (Figure 7d), confirming its reliability in this particular application scenario; see Section S9 and Figure S10 for detailed data. However, the MDLF model performs significantly better than the LMP model, with an average *R^2^* value of 0.92.

There are essential differences between the two models in the core logic: The LMP model treats the material response through an empirical stress‐time‐temperature relation and does not explicitly account for microstructural heterogeneity. In contrast, the MDLF can use microstructural micrographs to capture performance variations associated with structural heterogeneity, whereas the LMP baseline does not explicitly include microstructural information.

To verify the impact of the multimodal attention mechanism, the study compared the framework against a standard concatenation‐based benchmark (Section S10). Figure S11 presents the outcome, showing that although the concatenation model surpassed the unimodal versions with an average *R* ^2^ of 0.86, it did not match the accuracy of the attention‐based MDLF, which reached an average *R* ^2^ of 0.92. This difference confirms that the attention mechanism functions as a critical driver for information synthesis. It enables effective deep feature integration where simple concatenation fails.

The improvement of MDLF framework prediction performance is derived from a data fusion strategy that is substantially different from the standard splicing method. The traditional method simply stacks feature vectors into a linear sequence, which by default gives the same weight to all inputs, meaning that the burden of distinguishing useful signals falls on the downstream connected layer and this setting affect the efficiency of the optimization process. In contrast, the attention mechanism proposed in this paper adopts an active fusion method and uses process parameters as dynamic query to guide image feature analysis. This process prioritizes the selection of critical visual features, focuses on the morphological features of the delta phase and minimizes interference from background noise. Such an attention mechanism yields an analytical focus that is highly analogous to the targeted analysis carried out by domain experts. Instead of relying on simple additive combinations, the system produces context‐specific feature representations. As such, this selective attention is the key reason behind the improved prediction accuracy.

**FIGURE 8:**
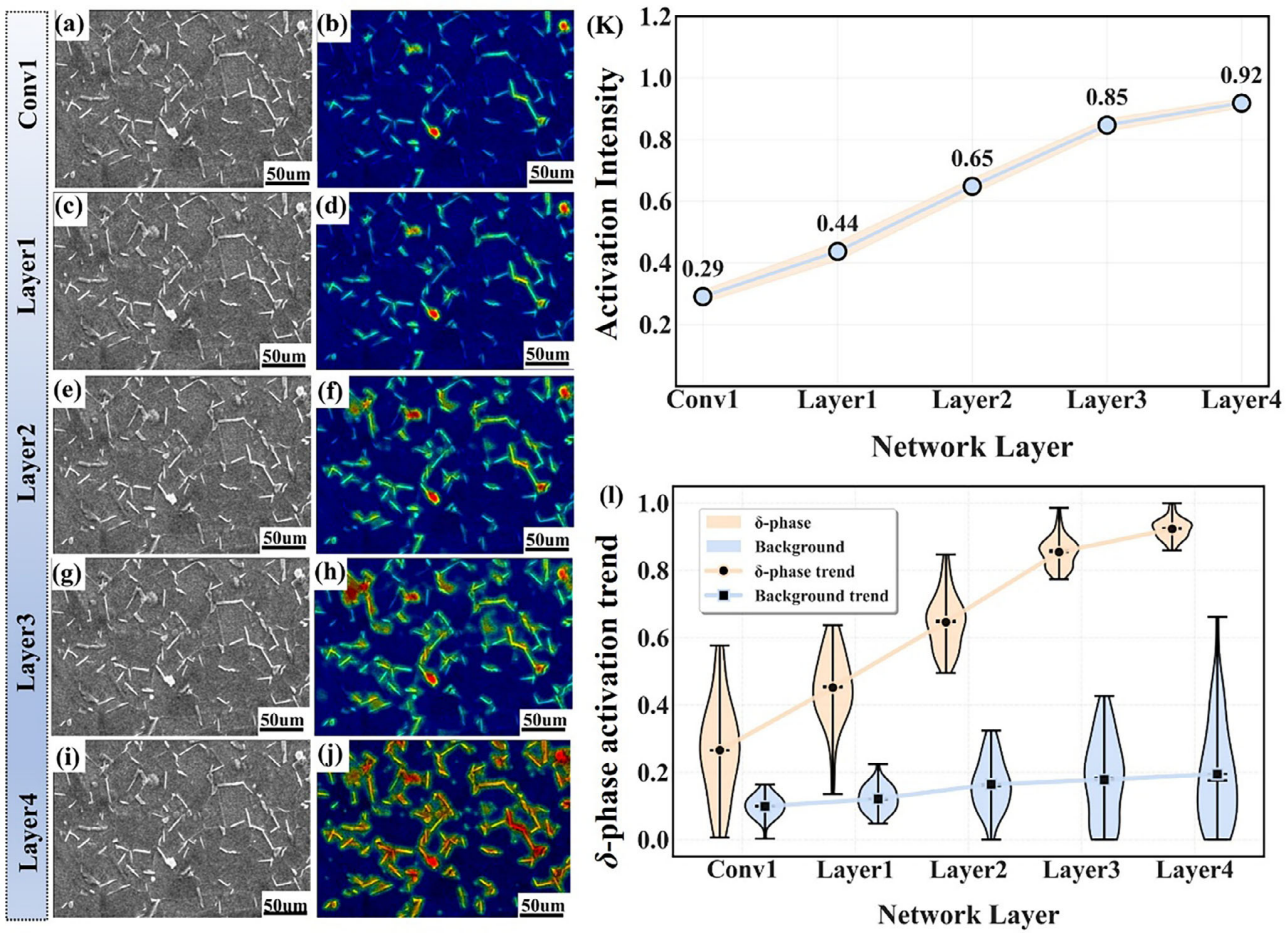
Evolution of the MDLF backbone feature representation at 980°C. (a, c, e, g, i) Original SEM micrographs; (b, d, f, h, j) Corresponding activation maps extracted from Conv1 and Layer 1 to Layer 4. These maps represent the normalized feature activations obtained through channel aggregation. (k) Quantitative comparison of the average normalized activation values across different network layers. (l) Violin plots showing the hierarchical evolution of the activation intensity distribution for the δ‐phase and background regions.

The step‐by‐step learning of the model is shown in Figure 8a–j. In the first network layers (Conv1, Layer 1), the initial layers extract basic visual information, including the edges of particles and the boundaries of phases and the heat maps for these early layers show a scattered focus (Figure 8a–d), with bright spots that are disconnected and point to simple edges and boundaries. In the middle layers of the network (Layer 2, Layer 3), these basic features start to come together, beginning to form more organized shapes (Figure 8f,h) and the focus then shifts to the actual shape and structure of the δ‐phase. In the last layer (Layer 4), the feature representation is finalized (Figure 8j): the highest activation values are concentrated on the δ‐phase regions while ignoring the background. This indicates that the model has established a robust, high‐level hierarchical representation of the critical microstructural features.

This definitive observation is quantitatively supported by the statistical analysis shown in Figure 8k,l. The average brightness of the delta phase region increases steadily with increasing network layers (Figure 8k), which improves the ability of the model to prioritize this key feature. The violin plot (Figure 8l) further illustrates the evolution of the feature activation distribution: for the δ‐phase feature, the activation value continues to rise, becoming clearly distinguishable from the background region, which maintains a lower level of activation. The most significant increase in activation values occurs between Layer 2 and Layer 3, indicating that this stage serves as a critical node where the model progressively isolates and resolves the key microstructural phase. The most significant increase in attention values occurs between layers 2 and 3, indicating that this phase is the critical node for achieving fusion of material characteristics.

Significantly, this highly effective, domain‐specific feature learning is a direct consequence of the end‐to‐end multimodal training strategy. Although the multimodal attention mechanism operates on the final, pooled feature vector, the gradients training the objective are backpropagated throughout the entire network. This process continuously refines the convolutional filters in all layers, compelling them to become highly selective for microstructural features that are most correlated with the tabular processing data. This enables a selective enhancement of the process‐microstructure coupling to emerge even in early and intermediate layers. Therefore, the visualization results collectively indicate that the multimodal guidance confers a domain‐specific sensitivity that surpasses conventional image‐only learning, aligning the internal analytical workflow with that of materials research.

Although the dominant strengthening mechanism of IN718 is governed by the nanoscale γ′ and γ′′ precipitates, these features cannot be resolved from the SEM images employed in the present study. However, their evolution is associated with mesoscale microstructural characteristics, including δ‐phase morphology, grain‐boundary decoration, and the corresponding heat‐treatment history. Therefore, the proposed multimodal framework combines compositional information, which provides thermodynamic constraints related to the underlying precipitation state, with morphological information that captures its mesoscale structural manifestation. Although the two modalities contain partially overlapping information, their integration provides complementary descriptions across different structural scales and compensates for the absence of high‐throughput nanoscale characterization (e.g., TEM). Consequently, the framework does not directly identify individual γ′ or γ′′ precipitates but instead exploits correlated mesoscale features as indirect indicators of their evolution. A more detailed discussion of this multiscale information complementarity is provided in Section S11.

**FIGURE 9:**
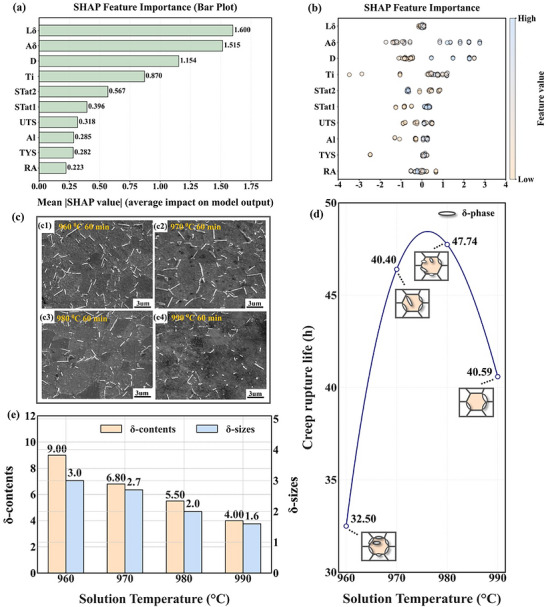
Descriptor‐based post hoc SHAP interpretation of microstructural factors associated with MDLF predictions. (a) SHAP bar plot ranking features by mean absolute SHAP; L*_δ_*, A*_δ_*, D dominate. (b) SHAP summary plot showing feature weights and positive/negative prediction impacts. (c) IN718 SEM images: needle δ‐phase dissolves with rising 960°C–990°C solution temperature. (d) Non‐monotonic creep life curve peaking at 980°C; insets show δ‐phase effects. (e) Plots of δ‐phase fraction and size vs. solution temperature.

To facilitate physical interpretation of the MDLF predictions, a Random Forest model was used to connect the trained MDLF with measurable microstructural descriptors. The MDLF was trained using alloy composition, heat‐treatment parameters, and SEM microstructural images, whereas δ‐phase volume fraction, δ‐phase size, and grain size were not used as explicit numerical inputs. Although the MDLF learns image‐based representations relevant to δ‐phase morphology and grain‐boundary features, these latent representations are high‐dimensional and cannot be directly expressed as quantitative metallurgical descriptors. Therefore, after MDLF training and validation, quantitative microstructural descriptors were extracted from the corresponding SEM/OM images using image analysis procedures. The Random Forest model used these descriptors as input variables and the corresponding MDLF predictions as target outputs. In this way, the model reveals the prediction behavior of the trained MDLF in a physically interpretable descriptor space. SHAP analysis was performed on the model to evaluate which microstructural descriptors were associated with the MDLF prediction behavior. Figure 9a,b presents the SHAP importance ranking obtained from the Random Forest model. Elucidating the underlying mechanisms of these key features facilitates a deeper understanding of the complex interactions among material composition, microstructure, and creep rupture life in the IN718 alloy. Given that the multimodal data fusion model emphasizes image‐derived features, this study concentrates on the effects of the top three factors on creep rupture life. A detailed explanation of the SHAP features is provided in Section S12.

The δ‐phase volume fraction, size, and matrix grain size are key microstructural factors governing the creep rupture life of the IN718 alloy. Under lower solution treatment temperatures (960°C), the presence of a high‐volume fraction of δ‐phase with predominantly fine, acicular morphology offers limited resistance to grain boundary sliding, resulting in relatively short creep rupture life. SHAP analysis corroborates this physical mechanism as feature values associated with this state, specifically small L*_δ_* and high A*_δ_*, exert a complex and predominantly negative influence on predicted rupture life. In the intermediate temperature range, thermally activated diffusion drives the coarsening of the δ‐phase via Ostwald ripening [38, 39]. This process induces a morphological transition to coarse, blocky or short‐rod structures, which enhances grain boundary pinning and impedes dislocation motion. This microstructural stability leads to improved creep resistance and the peak creep rupture life seen in Figure 9c–e. The model extracted this relationship; the SHAP values attribute the primary positive contribution for life prediction to features associated with this coarsened state. This effectively confirms the model can recognize the optimal microstructural configuration for creep performance. Conversely, as temperatures approach the δ‐phase solvus (approximately 990°C–1020°C for IN718 [40, 41, 42, 43]), prevalence of dissolution kinetics over coarsening causes a marked reduction in both volume fraction and dimensions of the δ‐phase. Diminishment of the grain boundary pinning phase increases susceptibility to creep deformation and precipitates a rapid decline in creep rupture life, a degradation behavior reproduced by the model to indicate learning of a non‐linear relationship where the strengthening effect of the δ‐phase diminishes beyond a critical temperature threshold.

### 3.1 Implications and Future Outlook

Although the proposed MDLF framework demonstrates accurate prediction and physically meaningful interpretation, its applicability is currently bounded by both the experimental scope and the model itself. The present study is based on creep rupture data obtained under a fixed testing condition (650°C/690 MPa). Therefore, the framework predicts rupture‐life variations associated with alloy composition, heat treatment, and the resulting microstructural evolution within this experimental domain, rather than across different creep temperatures or applied stresses. More importantly, the primary contribution of this work is to establish a trustworthy multimodal learning strategy for data‐scarce materials systems through the fusion of processing information and microstructural images, rather than to develop a creep‐life prediction model. Future extension to multiple creep conditions will require additional datasets covering different temperature‐stress combinations, with creep temperature and applied stress incorporated as explicit model inputs. In addition, the current framework has been validated using IN718, where the dominant microstructural evolution is governed by δ‐phase precipitation. Its application to more complex multi‐phase alloy systems may require corresponding modifications to the feature extraction and multimodal fusion strategies. The potential transferability of the pretrained framework to other precipitation‐strengthened Ni‐based superalloys is briefly discussed in Section S13. Although Grad‐CAM, attention‐weight visualization and descriptor‐based SHAP analysis improve the physical interpretability of the model, they do not reveal the underlying causal mechanisms of microstructural evolution. Future work will therefore integrate additional characterization modalities, such as EBSD‐derived crystallographic and strain information, together with physics‐informed constraints to further improve the generality, physical consistency and interpretability of the framework.

## 4 Conclusions

A trustworthy multimodal fusion framework, centered on a multimodal attention mechanism was developed for the accurate prediction of heat‐treatment‐dependent creep rupture life in IN718 alloy. The framework demonstrates high predictive accuracy and robustness on a limited dataset by synergistically integrating microstructural images with processing data, presenting a practical multimodal learning strategy. The main conclusions are as follows:

1. The adoption of a multimodal fusion strategy, centered on a multimodal attention mechanism, fundamentally overcomes the limitations of single‐modality approaches. The framework demonstrates a superior ability to interpret microstructural images, achieving excellent test performance (R2 = 0.92) by effectively capturing the critical microstructural characteristics associated with the δ‐phase, including its morphology and its non‐local interaction with the surrounding matrix.
2. The framework successfully captures the complex, non‐linear influence of the heat‐treatment path on creep rupture life. Visual interpretability analysis confirms that the model predictions align strongly with experimental observations, revealing the underlying physical trends with high fidelity.
3. The descriptor‐based post hoc SHAP analysis performed on the Random Forest model provides quantitative evidence for the critical role of δ‐phase characteristics in governing the creep mechanism. By ranking the contributions of key features, the analysis elucidates intrinsic structure‐property relationships and offers important mechanistic guidance for future ML‐driven materials design.

## 5 Methods

The detailed methods for data collection, processing, and model evaluation are presented in Section S14 (including Tables S2–S5 and Figure S13). All experimental data used for model development were obtained under a fixed creep testing condition (650°C/690 MPa). Therefore, creep temperature and applied stress were not treated as variable model inputs in this study.

## Author Contributions

**Jie Kang**: supervision, data curation. **Qian Wang**: investigation, validation. **Haikun Ma**: formal analysis, project administration. **Ziyuan Rao**: writing – review and editing, conceptualization, methodology. **Dayong Wu**: methodology, writing – review and editing, conceptualization, visualization. **Wang Li**: software, formal analysis. **Haopeng Lv**: conceptualization, methodology, writing – review and editing, writing – original draft, visualization. **Huicong Dong**: formal analysis, validation. **Zhinan Yang**: resources, data curation, investigation. **Ru Su**: writing – review and editing, resources, funding acquisition. **Yandong Wang**: validation, supervision, resources.

## Conflicts of Interest

The authors declare no conflicts of interest.

## Supporting information




**Supporting File**: advs77026‐sup‐0001‐SuppMat.docx.

## Data Availability

Data and original code used for training/testing the ML model, validation of the model, and formal analysis have been deposited at the GitHub repository: https://github.com/lvhaopeng11‐droid/‐MDLF‐for‐creep‐life‐.git. Due to file size limitations imposed by GitHub for image hosting, all associated image data, including the training, validation, and test sets are deposited in a dedicated large‐data repository (maintained by our project team) accessible via: https://github.com/lvhaopeng11‐droid/‐deep‐learning‐framework‐for‐creep‐life‐.git. Visualizations of the attention mechanisms presented in the manuscript can be accessed at: https://github.com/lvhaopeng11‐droid/‐MDLF‐Visualization.git. These are publicly available as of the date of publication. Further inquiries are welcome by the lead contact.
