# Supplementary material for: Trustworthy Multimodal Attention Framework for Creep Rupture Life Prediction Under Data‐Scarce Conditions: A Case Study on IN718
Source: Adv Sci (Weinh). 2026 Aug 10:e77026. Online ahead of print. doi: 10.1002/advs.77026 (PMC13455174; doi:10.1002/advs.77026)
Supplement: Supplementary file 1 [file ADVS-9999-e77026-s001.docx]

**Trustworthy multimodal attention framework for creep rupture life prediction under data-scarce conditions: a case study on IN718**

Haopeng Lv^1^, Dayong Wu^1*^, Ziyuan Rao^2[1]*^, Qian Wang^1^, Haikun Ma^1^, Jie Kang^1^, Wang Li^1^, Huicong Dong^1^, Yandong Wang^3^, Zhinan Yang^4,5*^, Ru Su^1,3*^

*^1^ Hebei Short Process Steelmaking Technology Innovation Center, School of Materials Science and Engineering, Hebei University of Science and Technology, Shijiazhuang, 050018, China*

*^2^ National Engineering Research Center of Light Alloy Net Forming, Shanghai Jiao Tong University, Shanghai, 200240, PR China*

*^3^ State Key Laboratory for Advanced Metals and Materials, University of Science and Technology Beijing, Beijing, 100083, China*

*^4^ Hebei lron and Steel Laboratory, North China University of Science and Technology, Tangshan 063210, China*

5 *Yanzhao lron and Steelloboratory Tangshan 063210 China*
1. **Transfer learning model**

**Figure S1.** Overall workflow of Transfer Learning.

In this study, we use ResNet-18 as the backbone network for image feature extraction to process 134 experimental samples together with their associated tabular data, including alloy composition and heat-treatment parameters. The present framework was developed and evaluated using data collected under a fixed creep testing condition (650 ^o^C/690 MPa). After sample-level partitioning and subsequent image preprocessing, the final image-tile dataset comprised 1596 processed image tiles, distributed across the training, validation and test subsets as 1128, 240 and 228 tiles, corresponding to 94, 20 and 20 physical samples, respectively. Large-scale models such as ResNet-18 were originally designed for millions of images and face pronounced overfitting risks when applied to datasets with limited numbers of unique samples. Although cropping and augmentation expand the apparent dataset size, this numerical increase does not fundamentally raise the number of distinct microstructure-property relationships available for learning. To address this challenge, we exploit the hierarchical feature-learning capability of ImageNet-pretrained ResNet-18 to effect efficient knowledge transfer to materials-science applications.

This study adopts a systematic transfer-learning approach that fully exploits the hierarchical feature-learning capacity of a pretrained ResNet-18. Specifically, the pretrained ResNet-18 is decomposed into a functionally independent feature extractor (convolutional layers conv1-layer4) and a classifier (fully connected layer); the extractor is retained intact while the original classifier is replaced with a custom multimodal fusion module, so that the modified backbone outputs a 512-dimensional feature vector via global average pooling, completely removing any dependence on the ImageNet classification head. Building on the principle of representational hierarchy, we observe that the ResNet-18 convolutional stack learns transferable features at distinct abstraction levels: low-level features (stages 1-2) encode generic visual primitives such as edge detection and texture patterns; mid-level features (stage 3) capture composite textures and local structural motifs and high-level features (stage 4) can be fine-tuned to material specific patterns such as δ-phase morphology and grain-boundary characteristics providing a theoretical basis for effective cross-domain knowledge transfer. The ResNet-18 backbone was initialized using ImageNet-pretrained weights. During model training, the shallow convolutional layers were kept frozen to preserve generic low-level feature extractors, while the deeper convolutional layers and multimodal regression components were trained for material-specific adaptation. The model was optimized using AdamW with an initial learning rate of 1×10^-3^), weight decay of 1×10^-4^) and a ReduceLROnPlateau scheduler. Early stopping with a patience of 40 epochs was applied based on the validation loss. Thus, the final training duration was determined by validation performance rather than by a fixed 100-epoch schedule.

The regularization scheme included dropout in the tabular embedding layer (0.2), dropout in the regression head (0.3), weight decay of 1×10^-4^), early stopping with a patience of 40 epochs and gradient clipping with a maximum norm of 1.0.

Despite substantive semantic differences between natural and microstructural images, the underlying visual elements exhibit striking consistency. Microstructural images contain edges (grain and phase boundaries), textures (surface relief and crystallographic orientation), contrast variations (compositional differences) and spatial arrangements (phase distributions), all of which map onto the visual primitives learned from ImageNet. This structural homology enables effective transfer of low and mid-level features, requiring high-level adaptation for material-specific pattern recognition.

2. **Deep learning model**
   2.1 **Query-Key-Value assignment scheme**

The direction of cross-modal information aggregation is determined by the assignment of the Query, Keys and Values. In the implemented MDLF, the tabular/process representation serves as a single Query token, whereas the spatial image representations serve as the Keys and Values.

The tabular branch provides a sample-level representation of alloy composition and heat-treatment conditions. The image branch provides 49 spatial representations derived from the 7×7 Stage-4 feature map. Therefore, the selected assignment allows the tabular/process state of each specimen to condition the aggregation of spatially distributed image information.

Reversing the assignments would not produce an equivalent operation. If the 49 image tokens were used as Queries and the tabular representation were used as a single Key and Value, the module would produce 49 output tokens. Moreover, the Softmax operation over a single tabular Key would yield a weight of one for every image Query, eliminating competition among spatial locations. The implemented assignment was retained because it produces one sample-level fused representation by weighting 49 image tokens under the corresponding tabular/process context.

2.2 **Query-Key-Value setting process**

To fuse tabular and image features, we introduce a multimodal attention mechanism in our model. Specifically, the tabular embedding serves as the Query, whereas the spatial image tokens serve as the Key and Value. The attention module is applied to the Stage-4 spatial feature map extracted from the ResNet-18 backbone to generate a tabular-conditioned visual representation for subsequent regression.

1) The normalized tabular data are encoded by a two-layer MLP to obtain a 128-dimensional embedding, which serves as the Query. The tabular embedding is denoted as $Z_{table}\in R^{B\times D}$, where $B$is the batch size and $D$=128 is the embedding dimension.

2) The spatial feature map extracted from the final convolutional block of ResNet-18, i.e., Stage 4, was used as the image-token representation for multimodal attention. This feature map is denoted as $F_{4}\in\mathbb{R}^{B\times512\times7\times7}$. After flattening the spatial dimensions, it becomes $F_{\mathrm{tok}}\in\mathbb{R}^{B\times49\times512}$. These image tokens are projected to generate the Key and Value representations. The linear projections and multi-head attention operation are shown in Equations S1-S4. The residual fusion and layer normalization are shown in Equation S5.

$Q=(Z_{table})W_{q}$	(S1)

$K=F_{tok}W_{k}$	(S2)

$V=F_{tok}W_{v}$	(S3)

Here, $Z_{table}\in\mathbb{R}^{B\times D}$ denotes the 128-dimensional tabular embedding obtained by the two-layer MLP and $W_{q}$ projects it into the query space. To perform batched matrix multiplication in the attention mechanism, the resulting query tensor is mathematically treated as a single-token sequence, effectively expanding its shape to *B×1×D* for the $h$-th head). $F_{tok}$denotes the flattened Stage-4 image tokens obtained from $F_{4}$, whereas $W_{k}$and $W_{v}$project the image tokens into the key and value spaces, respectively. For the subsequent multi-head attention operation, the tabular embedding is treated as a single Query token, whereas $F_{tok}$contains 49 spatial image tokens.

$Attn\left( Q,K,V \right)={Concat}_{h=1}^{H}{[{Softmax}_{N}(\frac{Q_{h}K_{h}^{T}}{\sqrt{d_{h}}})V_{h}]W}_{o}$	(S4)

$F_{fused}=LN[Z_{table}+Dropout\left( Attn\left( Q,K,V \right) \right)]$	(S5)

Here, $H$=8 denotes the number of attention heads, $D$=128denotes the total attention dimension and $d_{h}=\frac{D}{H}=$16denotes the feature dimension of each attention head. $N=$49denotes the number of spatial image tokens obtained by flattening the 7×7Stage-4 feature map. For the $h$-th attention head, $Q_{h}\in\mathbb{R}^{B\times d_{h}}$is derived from the tabular/process embedding, whereas $K_{h},V_{h}\in\mathbb{R}^{B\times N\times d_{h}}$are derived from the spatial image tokens.

The scaled dot product $\frac{Q_{h}K_{h}^{T}}{\sqrt{d_{h}}}$ produces compatibility scores between the tabular/process Query and the $N$spatial image Keys. The scaling factor $\sqrt{d_{h}}$prevents the magnitude of the dot products from increasing excessively with the feature dimension. The Softmax operation is applied along the image-token dimension $N$, yielding normalized attention weights over the 49 spatial image tokens. For each sample and attention head, these attention weights sum to one.

Multiplication of the normalized attention weights by $V_{h}$produces a weighted aggregation of the spatial image representations. The outputs of the eight attention heads are concatenated along the feature dimension and transformed by the output projection matrix $W_{O}\in\mathbb{R}^{D\times D}$. Consequently, $\mathrm{Attn} \left( Q,K,V \right)\in\mathbb{R}^{B\times D}$. The projected attention output is subjected to dropout and combined with the original tabular/process embedding $Z_{\mathrm{table}}\in\mathbb{R}^{B\times D}$ through a residual connection. Layer normalization is applied along the feature dimension to improve optimization stability and produce the tabular-conditioned fused representation $F_{\mathrm{fused}}\in\mathbb{R}^{B\times D}$ where $D=$128. In parallel, global average pooling is applied to the Stage-4 image feature map to obtain a 512-dimensional image-level representation, $F_{\mathrm{global}}=\mathrm{GAP}(F_{4})\in\mathbb{R}^{B\times512}$. The final multimodal representation is formed by concatenating the global image representation and the tabular-conditioned fused representation:$F_{\mathrm{final}}=Concat$($F_{global},F_{fused}$)$\in\mathbb{R}^{B\times640}$. The resulting 640-dimensional feature vector is passed to the final regression head for creep rupture life prediction.

The detailed pseudocode is as follows:

\# [B, N, C], where N = H × W

\# Query from tabular embedding

Q = z_tab @ W_q + b_q	# [B, C]

Q = Q.view(B, h, d_h)	# [B, h, d_h]

\# Keys and Values from image feature tokens

K = F_flat @ W_k + b_k	# [B, N, C]

V = F_flat @ W_v + b_v	# [B, N, C]

K = K.view(B, N, h, d_h)	# [B, N, h, d_h]

V = V.view(B, N, h, d_h)	# [B, N, h, d_h]

\# Scaled dot-product attention per head

O_heads = []

for j in range(h):

Qj = Q[:, j, :].unsqueeze(1)	# [B, 1, d_h]

Kj = K[:, :, j, :] # [B, N, d_h]

Vj = V[:, :, j, :] # [B, N, d_h]

scores = (Qj @ Kj.transpose(1, 2)) / (d_h ** 0.5)	# [B, 1, N]

A = torch.softmax(scores, dim=-1)	# [B, 1, N]

Oj = A @ Vj	# [B, 1, d_h]

O_heads.append(Oj)

\# Concatenate multi-head outputs

O = torch.cat(O_heads, dim=-1)	# [B, 1, C]

O = O.squeeze(1)	# [B, C]

\# Output projection and dropout

O = dropout(O @ W_o + b_o)	# [B, C]

\# Residual connection + LayerNorm

z_fused = layer_norm(z_tab + O)	# [B, C]

\# Final feature concatenation for regression

z_final = torch.cat([image_global, z_fused], dim=-1)

\# Regression prediction

y_pred = regression_head(z_final)

**Table S1.** Details of the MDLF architecture used in this work

| **Layer(type)** | **Filter size/#of filters** | **Key Parameters** | **Output Shape(W×H×C)** |
| --- | --- | --- | --- |
| Input | - | - | 224×224×3 |
| Stem-Conv | 7×7/64/s=2 | padding=3,BN,ReLU | 112×112×64 |
| Stem-MaxPool | 3×3/s=2 | padding=1 | 56×56×64 |
| Stage1-Block1-Conv1 | 3×3/64/s=1 | padding=1,BN,ReLU,Frozen | 56×56×64 |
| Stage1-Block1-Conv2 | 3×3/64/s=1 | padding=1,BN,Frozen | 56×56×64 |
| Stage1-Block2-Conv1 | 3×3/64/s=1 | padding=1,BN,ReLU, Frozen | 56×56×64 |
| Stage1-Block2-Conv2 | 3×3/64/s=1 | padding=1,BN,Frozen | 56×56×64 |
| Stage2-Block1-Conv1 | 3×3/128/s=2 | padding=1,BN,ReLU,Frozen | 28×28×128 |
| Stage2-Block1-Conv2 | 3×3/128/s=1 | padding=1,BN,Frozen | 28×28×128 |
| Stage2-Block1-Downsample | 1×1/128/s=2 | BN, Frozen | 28×28×128 |
| Stage2-Block2-Conv1 | 3×3/128/s=1 | padding=1,BN,ReLU,Frozen | 28×28×128 |
| Stage2-Block2-Conv2 | 3×3/128/s=1 | padding=1,BN,Frozen | 28×28×128 |
| Stage3-Block1-Conv1 | 3×3/256/s=2 | padding=1,BN,ReLU,Trainable | 14×14×256 |
| Stage3-Block1-Conv2 | 3×3/256/s=1 | padding=1,BN,Trainable | 14×14×256 |
| Stage3-Block1-Downsample | 1×1/256/s=2 | BN, Trainable | 14×14×256 |
| Stage3-Block2-Conv1 | 3×3/256/s=1 | padding=1,BN,ReLU,Trainable | 14×14×256 |
| Stage3-Block2-Conv2 | 3×3/256/s=1 | padding=1,BN,Trainable | 14×14×256 |
| Stage4-Block1-Conv1 | 3×3/512/s=2 | padding=1,BN,ReLU,Trainable | 7×7×512 |
| Stage4-Block1-Conv2 | 3×3/512/s=1 | padding=1,BN,Trainable | 7×7×512 |
| Stage4-Block1-Downsample | 1×1/512/s=2 | BN, Trainable | 7×7×512 |
| Stage4-Block2-Conv1 | 3×3/512/s=1 | padding=1,BN,ReLU,Trainable | 7×7×512 |
| Stage4-Block2-Conv2 | 3×3/512/s=1 | padding=1,BN,Trainable | 7×7×512 |
| Global Average Pool | 1×1 | - | 1×1×512 |
| Image Feature Flatten | - | - | 512 |
| **Tabular Branch** | - | - | - |
| Tabular Input | - | Normalized features | n_features |
| Tabular Embedding FC1 | n_features-128 | BN, ReLU, Dropout=0.2 | 128 |
| Tabular Embedding FC2 | 128-128 | BN, ReLU | 128 |
| **Cross-Modal Attention Fusion** | - | - | - |
| Image Feature Projection | 512-128 | Linear transform for K,V | 128 |
| Multi-Head Attention | 8 heads | Q=tabular(128),k=V=image(128) | 128 |
| Attention Output Projection | 128-128 | Linear projection,LayerNorm | 128 |
| Residual Connection | - | Tabular+Attention output | 128 |
| Feature Connection | - | Concat(image_512,fused_128) | 640 |
| **Regression Head** | - | - | - |
| FC1 | 640-256 | ReLU,Dropout=0.3 | 256 |
| FC2 | 256-128 | ReLU,Dropout=0.3 | 128 |
| Output Layer | 128-1 | linearly | 1 |

3. **Validation framework and process**

**Figure S2.** Validation framework used in this study.

**Figure S3.** Test-set *R^2^* across 50 random-seed training repetitions under the fixed primary specimen partition.

The primary evaluation was based on one predefined composition-stratified split of 134 physical specimens into 94 training, 20 validation and 20 test samples. All images, image tiles, tabular variables and target values belonging to the same specimen were assigned to the same subset. The partition remained fixed across 50 random-seed training repetitions.

The mean test-set *R^2^* was 0.917, with a standard deviation of 0.014 and a 95% confidence interval for the mean of [0.913, 0.921] (Figure S3). The corresponding mean *RMSE* and *MAPE* were 0.14 and 6.0%. The standard deviation and confidence interval quantify training stochasticity under this fixed specimen partition and should not be interpreted as uncertainty associated with repeated random data splitting.

**Figure S4.** Predictive performance of the MDLF model across the Training, Validation and Test sets.

4. **Five-fold cross-validation process**

The Five-fold cross-validation was conducted as a supplementary assessment of sensitivity to specimen partitioning. The 134 physical specimens were assigned to five mutually exclusive folds using composition-stratified grouping. All data derived from a specimen remained in the same fold.

In each outer iteration, one-fold was retained as the test fold. A validation subset was selected from the remaining specimens and was used for early stopping. Image normalization, tabular normalization and any other data-dependent preprocessing were fitted using the training specimens of that iteration. The outer test fold was not used for model selection, early stopping, and preprocessing. These values provide supplementary evidence concerning partition sensitivity and are not the source of the headline result reported in the Abstract.

As shown in Figures. S5a-c, the average performance across the five validation runs confirms that the model is both robust and stable, with an average *R^2^* of 0.92 and an average *RMSE* of 0.18. This result suggests that the MDLF performance is not dependent on the specific predefined split and that the model can capture useful process-structure-property relationships from the limited dataset.

To understand the unexpected performance from the initial data split, we compared the distributions of key features between the training and test sets. These features are critical to creep rupture life and include δ-phase volume fraction (A*_δ_*), δ-phase size (L*_δ_*) and grain size (D). The kernel density estimation (KDE)^[1]^ plots in Figures. S5d-f show that these distributions were different. The samples in the initial test set exhibited a more centralized distribution on the critical characteristics (L*_δ_* and D). In contrast, the training set covered a much broader spread of data, including more extreme and difficult cases. This suggests that the initial test set inadvertently contained samples with simpler, more dominant data patterns. Therefore, the observed performance anomaly is attributed to a statistical artifact of random sampling in a small dataset, rather than an inadequacy of the model itself. Combined with the robust performance confirmed by cross-validation, these findings affirm the capability of MDLF to model complex material property relationships effectively.

**Figure S5.** Five-fold cross-validation was performed to validate the MDLF framework. Feature space distributions were also examined. a) A parity plot compares predicted values against experimental data. The data points display the agreement between log-transformed creep rupture life derived from cross-validation and the measured creep rupture life. b) Overall performance metrics of the model on the training and validation sets. This 3D bar chart displays the average coefficient of *R^2^* and RMSE, showing an *R^2^* of 0.92 and an RMSE of 0.18. c) Performance metrics for each individual fold in the cross-validation. This chart illustrates the *R^2^* and RMSE values across the five different data subsets and the consistent results demonstrate the model's robustness. d), e), f) Comparison of the distributions of key microstructural features between the training and testing sets. These histograms compare the distributions of d) average δ-phase volume fraction, e) average δ phase size and f) average grain size, respectively. The similar distributions ensure a fair data split and support the generalization capability of the model.

5. **Model uncertainty evaluation**

**Figure S6.** Uncertainty quantification (UQ) of model predictions using Monte Carlo Dropout (MC Dropout). a) Displays the probability density histogram of the creep rupture life predictions for a single sample (true value 10.3h) based on 100 random forward propagations (T=100). The figure includes the fitted log-normal distribution curve, the predicted mean (14.0h) as the final prediction, and the 95% confidence interval representing the uncertainty; b) is the prediction vs. true value comparison plot (Parity Plot) with uncertainty error bars.

To quantitatively evaluate the model’s prediction confidence, we plan to use the Monte Carlo Dropout (MC Dropout) technique, which is an efficient method for Bayesian approximate inference in deep learning. It transforms the Dropout layers, originally used for overfitting prevention, into a powerful tool for uncertainty estimation during the inference phase. The core principles and implementation details are outlined below:

Unlike the standard practice of completely disabling the Dropout layers during inference (which makes the network deterministic), the MC Dropout method intentionally keeps the random activation of the Dropout layers during the prediction phase. For a given sample to be tested (which includes its microstructural image and tabular process parameters), we no longer perform a single, deterministic forward pass. Instead, we perform $T$ independent stochastic forward passes (Stochastic Forward Passes), for example, setting $T$=100. In each forward pass, the Dropout layers in the network randomly set a portion of the neuron outputs to zero with a preset probability. This is equivalent to performing random sampling from the original fully connected network, resulting in a unique "sub-network". Therefore, $T$ independent stochastic forward passes are equivalent to sampling $T$ times from an implicit model ensemble consisting of many sub-networks, resulting in $T$ slightly different creep rupture life predictions, denoted as {*y__1_, y__2_, ..., y__T_*}.

This set of $T$ prediction values no longer represents a single point estimate but forms an empirical posterior distribution regarding the prediction results. The mean of this empirical distribution is used as the final prediction for creep rupture life. As the integrated average of $T$ different sub-network predictions, this mean is typically more robust and accurate than the result of any single deterministic forward pass, effectively smoothing out the potential biases of the individual model. Furthermore, the standard deviation of the empirical distribution is used as a direct quantification of the model's epistemic uncertainty. This uncertainty stems from the model's own knowledge limitations, meaning that due to the finite training dataset, the model parameters have not perfectly converged to an optimal solution, but rather exist within a confidence range. Therefore, a larger standard deviation suggests that when faced with the current input sample, the numerous "sub-networks" within the model give predictions with significant divergence, strongly indicating that the sample may lie in an out-of-distribution region, where the training data coverage is insufficient. The uncertainty evaluation described above offers a quantitative, dynamic confidence assessment, enabling the model to possess genuine "self-awareness."

Notably, the uncertainty estimated by Monte Carlo Dropout should not be interpreted using a universal numerical threshold because the acceptable confidence level depends on the engineering objective, risk tolerance and decision scenario. For example, when the framework is used for preliminary high-throughput alloy screening, relatively larger predictive uncertainty still be acceptable because candidate materials will undergo subsequent experimental validation. In contrast, when the prediction is intended to support engineering design and service-life assessment of critical structural components, a more conservative uncertainty criterion should be adopted.

Therefore, the uncertainty estimated by the proposed framework should be regarded as a decision-support indicator rather than an absolute acceptance criterion. In practical applications, acceptable uncertainty thresholds should be determined according to engineering requirements, safety margins, experimental cost and the consequences associated with prediction errors. Future work will investigate application-specific uncertainty criteria through large-scale engineering validation.

6. **Visualization of multimodal attention weights**

**Figure S7.** Visualization of actual attention weights extracted from the multimodal attention module. The weights were computed from the softmax-normalized $QK^{T}$matrix, averaged across eight attention heads, reshaped to the 7×7 spatial image-token layout and upsampled to the original SEM image size. These maps visualize the spatial image tokens receiving higher weights under the corresponding tabular/process context and are distinct from Grad-CAM saliency maps. In the overlaid heatmaps, brighter yellow regions indicate higher learned attention weights, whereas darker regions represent lower weights.

To distinguish the learned cross-modal attention weights from Grad-CAM saliency maps, we visualized the attention weights extracted directly from the multimodal attention module. For the $h$-th attention head, the attention weights were computed as:

$A_{h}=Softmax(Q_{h}K_{h}^{T}/\sqrt{d_{h}})$	(S6)

where $Q_{h}$denotes the tabular/process query and $K_{h}$denotes the Stage-4 image-token key. The attention weights from the eight heads were averaged, reshaped to the 7×7 spatial token layout and upsampled to the original SEM image size. The resulting maps show how strongly each spatial image token is weighted under the corresponding processing/composition context.

In the stages of the network (Conv1 and Layer 1), the activation heatmaps are widely dispersed, highlighting high-frequency visual elements such as phase interfaces, grain boundaries and basic contrast edges. The corresponding representations retain a broad spectrum of the image texture, showing that the shallow layers focus primarily on generic spatial and topological primitives. As the feature extraction progresses through the intermediate stages (Layer 2 and Layer 3), these disconnected local boundaries assemble into larger, cohesive structural motifs. The activation heatmaps show a noticeable consolidation, with the focus progressively shifting away from the matrix background and narrowing down toward the spatial coordinates of the δ-phase precipitates.

At the deepest stage (Layer 4), the model establishes a highly concentrated semantic representation. The activation hotspots align precisely with the needle-shaped and blocky morphologies of the δ-phase, while the irrelevant matrix background is effectively suppressed (approaching zero activation, as shown by the darkened areas in the representation). This hierarchical transition from edge-level features to phase-level semantics provides strong evidence of the physical interpretability of the framework. Instead of exploiting spurious background shortcuts and training-set noise, the network utilizes its hierarchical representation to isolate the critical δ-phase. Since the morphology, distribution and grain-boundary pinning effect of the δ-phase are established metallurgical drivers of creep resistance in the IN718 alloy, this localized focus confirms that the deep learning model has captured physically consistent structural representations to support its life predictions.

These attention maps are different from Grad-CAM maps. Grad-CAM is a gradient-based saliency method computed from the final convolutional feature maps, whereas the attention maps are obtained from the softmax-normalized $QK^{T}$matrix inside the multimodal attention module. Therefore, the two visualizations are presented separately and should be interpreted as complementary but distinct forms of model interpretation.

7. **TablePFN model**

Recent advancements in tabular deep learning, particularly the TablePFN model, have demonstrated remarkable performance on small datasets. TabPFN (Tabular Prior-Data Fitted Network) represents a paradigm shift in tabular deep learning.^[2]^ Unlike traditional machine learning models (e.g., XGBoost, Random Forest) that learn patterns by iterating on a specific dataset via gradient descent, TabPFN is a foundation model based on the Transformer architecture. It is pre-trained on millions of diverse synthetic datasets using a meta-learning strategy. During inference, it performs "in-context learning," meaning it treats the entire training set as a prompt to predict the test samples in a single forward pass, without any further hyperparameter tuning. To rigorously assess whether the superior performance of our MDLF stems from the multimodal architecture and simply outperforms weak baselines, we benchmarked TablePFN against our framework using two distinct validation protocols.

The stratified generalization protocol was designed to evaluate model generalization under a more challenging data partition and was used as a supplementary analysis. It should not be interpreted as the validation protocol supporting the headline performance reported in the main manuscript. The dataset comprises 134 samples derived from 3 distinct alloy compositions subjected to varied heat treatment series. This structure creates inherent clusters in the feature space.

**Figure S8.** Evaluation of prediction reliability under different data splitting protocols and model architectures. a) Parity plot of predicted versus actual logarithm of creep rupture life under the Random Split protocol. b) Parity plot under the Stratified Generalization protocol, representing a pure tabular baseline (TabPFN). c) Comparative histogram of *R^2^* scores and Mean Absolute Percentage Error (MAPE) for the tabular model under varied scenarios.

When the dataset is randomly split, the training and test sets share identical alloy compositions and overlapping process windows. Under this setting, TablePFN achieves an average *R^2^* approaching 0.90. This suggests the model acts as a highly efficient "lookup table," memorizing the cluster-specific outcomes. While statistically impressive, this does not reflect the physical stochasticity of creep rupture, which typically exhibits 10-20% experimental scatter even under identical conditions.

To test the model's ability to generalize to unseen process conditions (a requirement for materials design), we employed the rigorous stratified splitting described in the Methods. Under this protocol, the performance of TablePFN drops to an average *R^2^* of approximately 0.75.

The discrepancy between 0.90 (interpolation) and 0.75 (generalization) highlights that pure tabular features are insufficient to capture the non-linear variability of creep life outside of memorized clusters. The MDLF achieves an average *R^2^* of 0.92 under the same rigorous protocol. This confirms that the incorporation of microstructural images provides the necessary "ground truth" of the material's physical state (e.g., actual δ-phase dissolution degree), enabling the model to correct predictions where tabular data is ambiguous.

8. **Bar chart comparing overfitting across models**

**Figure S9.** Prediction performance of independent baseline models based on explicit data segmentation on the training, validation, and test sets. a-c) Traditional machine learning models; d) TablePFN baseline model; e) Weakened CNN; f) Pure vision models. Performance is shown across 50 independent replicates; error bars represent standard deviation.

In scenarios with limited data, a typical manifestation of overfitting is a significant drop in performance between the training and test sets. As shown in Figure S9(d), the TablePFN model exhibits clear overfitting on tabular data, with its *R^2^* dropping from 0.88 on the training set to 0.75 on the test set. Similarly, the pure vision model (Figure S9 (f)) also drops from 0.83 on the training set to 0.73 on the test set. This indicates that relying solely on a single modality will inevitably lead to memory-based overfitting on this challenging dataset. The very compact error bars in Figure S9(f) (from 50 independent runs with different random seeds) indicate that the performance of image feature extraction can be attributed to the intrinsic properties of the architecture, rather than to a lucky random initialization.

9. **Larson-Miller Parameter baseline**

To provide a physics-based empirical reference for creep rupture prediction, a Larson-Miller Parameter (LMP) baseline was constructed. The LMP is defined as:

$P=T(C+{log}_{10}t_{r})$	(S7)

where $P$is the Larson-Miller parameter, $T$is the absolute temperature in Kelvin, $t_{r}$is the rupture life in hours and $C$is a material-dependent constant. The LMP baseline used in this study was calibrated using a separate creep rupture dataset containing 266 data points collected under multiple temperature and stress conditions. This calibration dataset was used to establish the empirical relationship between the Larson-Miller Parameter and applied stress. After calibration, the fitted LMP relation was applied to the fixed creep-testing condition used in the MDLF evaluation, i.e., 650 ^o^C and 690 MPa.

These data cover multiple creep temperatures and applied stresses and were used to fit the empirical stress-LMP relationship. Specifically, after determining $C$, the calculated LMP values were fitted as a function of applied stress:

$P=a_{0}+a_{1}{log}_{10}\sigma+a_{2}({log}_{10}\sigma)^{2}$	(S8)

where $\sigma$ is the applied stress. The fitted polynomial was then used to predict $P$for the test samples and the rupture life was calculated as:

${log}_{10}t_{r}=\frac{P}{T}-C$	(S9)

The fitted Larson-Miller constant was $C$=21.6. The fitted coefficients were $a_{0}$=1.5, $a_{1}$=3 and $a_{2}$=2.5. The test-set performance of the fitted LMP baseline was *R^2^*=0.84for ${log}_{10}t_{r}$ (as show in Figure S10).

The LMP baseline was constructed using the creep rupture dataset containing multiple temperature and stress conditions. It was used as a conventional physics-based empirical reference. Because the MDLF developed in this work focuses on heat-treatment-dependent creep rupture prediction under a fixed creep condition, the LMP baseline should not be interpreted as a strictly identical-input benchmark.

**Figure S10.** Performance of the Larson-Miller Parameter baseline. a) Training set; b) Test set.

10. **Comparative ablation study of the model**

**Figure S11.** A comparative ablation analysis validating the essential role of the multimodal attention mechanism. a) Quantitative comparison of model performance as measured by *R^2^*. b) Comparison between predicted and ground-truth values for single-modality models. c) Results from a simplified multimodal baseline employing feature concatenation.

At the heart of this contrast lies the observation that, while simple concatenation enables the model to access information from both modalities at once, it provides no explicit, dynamic scheme for cross-modal interaction. It treats all features uniformly, without prioritization and offloads to the downstream MLP the full burden of learning the intricate mapping between particular process parameters and salient microstructural characteristics. By contrast, the attention-based architecture we propose imposes a powerful inductive bias: treating the tabular inputs as Queries, it actively and selectively attends to the portions of the image representation most pertinent to that context, yielding a weighted fusion of features.

11. **Data redundancy explanation**

Within the domain of multimodal materials informatics, a perennial critique concerns the inherent information redundancy between processing parameters and microstructural observations, following the fundamental metallurgical principle that process dictates structure. We posit, however, that in data-scarce regimes where comprehensive multiscale characterization is restricted, such redundancy constitutes a strategic trade-off necessary for achieving physical completeness. The mechanical properties of superalloys are governed by a complex hierarchy of features spanning from the atomic and nanoscale, including elemental segregation and γ′/γ′′ precipitate coherency which remain invisible in standard SEM-to mesoscale attributes like grain boundary topology and δ phase distribution. Given that high-throughput Transmission Electron Microscopy (TEM) is not currently feasible for large datasets, our framework adopts a complementation strategy that utilizes tabular data as a critical numerical proxy for these hidden nanoscale factors. In this context, tabular features define the "intended" thermodynamic boundaries and chemical potentials, while the corresponding SEM images capture the "realized" topological state and stochastic heterogeneities, such as local phase agglomeration or incomplete dissolution.

The synergistic integration of these disparate modalities is operationalized through a multimodal attention mechanism that effectively reconciles overlapping signals. Rather than being hampered by redundancy, the attention module treats the tabular parameters as contextual queries to interrogate the microstructural images, thereby filtering out superfluous correlations and amplifying the complementary information hidden in the mesoscale features. This approach enables the model to infer a holistic representation of the alloy’s state, capturing the coupled effects of sub-resolution nanoscale chemistry and explicitly resolved mesoscale morphology. Consequently, the observed redundancy is not a statistical inefficiency, but rather a robust compensatory strategy. It provides a reliable pathway to approximate the complete Process-Structure-Property (PSP) chain in environments lacking atomic-scale characterization, ensuring that the model’s predictive power remains deeply grounded in the multi-scale physical reality of the superalloy.

12. **Clarification of SHAP features**

**Figure S12.** Schematic workflow of descriptor-based post hoc SHAP interpretation and model performance. a) The overall workflow framework. b) arity plot comparing the Random Forest surrogate outputs with the MDLF predictions. The dashed line represents ideal agreement. c) Evaluation metrics (*R^2^* and RMSE) on the training, validation and testing datasets.

It should be emphasized that δ-phase volume fraction, δ-phase size and matrix grain size were not used as explicit numerical inputs to the MDLF. The MDLF was trained using alloy-composition variables, heat-treatment parameters and SEM microstructural images. During both training and inference, the quantitative microstructural descriptors were not provided to the MDLF as additional tabular variables.

The image branch of the MDLF generates high-dimensional latent representations from the SEM images. These representations may contain information associated with phase morphology, phase distribution and grain-boundary characteristics. However, they cannot be directly interpreted as explicit numerical measurements of δ-phase volume fraction, δ-phase size and grain size. Therefore, a separate post hoc surrogate-analysis procedure was used to relate the trained MDLF predictions to measurable metallurgical descriptors. After MDLF training and prediction, quantitative microstructural descriptors were obtained from the corresponding SEM/OM images using the image-analysis procedures described in the experimental section. The descriptor vector for sample $i$can be represented as:

$\boldsymbol{x}_{desc}^{\left( i \right)}=\left[ {X_{tab}^{(i)}, A}_{\delta}^{\left( i \right)},L_{\delta}^{\left( i \right)},D^{\left( i \right)} \right]$	(S10)

where $X_{tab}^{(i)}$ represents the explicit tabular variables of sample $i$ (including the 10 alloy-composition variables and 6 heat-treatment parameters, such as Ni, Co, Cr, Fe, Al, Ti, Nb, Mo, W, T, ST, STt, STat, Stat, AT, At). $A_{\delta}$, $L_{\delta}$ and $D$denote the δ-phase volume fraction, δ-phase size and matrix grain size, respectively. A Random Forest surrogate model, $g_{\mathrm{RF}}$, was trained using these post hoc descriptors as inputs. The target value for each sample was not the experimentally measured creep rupture life but the prediction generated by the trained MDLF:

$y_{target}^{\left( i \right)}=\hat{y}_{MDLF}^{\left( i \right)}$	(S11)

The surrogate output is expressed as:

$\hat{y}_{\mathrm{surrogate}}^{\left( i \right)}=g_{\mathrm{RF}}\left( \boldsymbol{x}_{desc}^{\left( i \right)} \right)$	(S12)

Thus, the Random Forest was not used as an independent creep rupture life predictor and was not part of the MDLF architecture. Its purpose was to approximate the prediction behavior of the trained MDLF in a lower-dimensional descriptor space that can be interpreted using conventional metallurgical quantities. The surrogate analysis was performed using a physical-sample-level train/validation/test partition. All descriptors and MDLF outputs belonging to the same physical sample were assigned to the same subset. The test samples were not used to fit the Random Forest surrogate.

Surrogate fidelity was evaluated by comparing $\hat{y}_{surrogate}$directly with $\hat{y}_{MDLF}$ The fidelity coefficient was calculated as：

$R_{fidelity}^{2}=1-\frac{\sum_{i} \left( \hat{y}_{MDLF}^{\left( i \right)} - \hat{y}_{surrogate}^{\left( i \right)} \right)^{2}}{\sum_{i} \left( \hat{y}_{MDLF}^{\left( i \right)} - {\bar{\hat{y}}}_{MDLF} \right)^{2}}$	(S13)

The Random Forest model achieved *R^2^*=0.94, 0.91 and 0.90 on the training, validation and test subsets, respectively. The corresponding *RMSE* values were 0.17, 0.15 and 0.12. In particular, the test-set *R^2^*=0.90 indicates that the descriptor-based model captures the principal variation in the MDLF predictions. SHAP analysis was applied to the Random Forest model. The resulting SHAP values quantify how each post hoc descriptor contributes to the model output relative to the model baseline. Therefore, these SHAP values describe the contributions of measurable microstructural descriptors to the model approximation of the MDLF predictions. They do not represent direct SHAP attributions of the original MDLF input variables.

Accordingly, a high SHAP importance for δ-phase volume fraction, δ-phase size and grain size does not mean that these quantities were explicitly supplied to the MDLF. Instead, it indicates that variation in the MDLF predictions can be substantially associated with variation in these descriptors after the high-dimensional prediction behavior is projected into the descriptor space. This descriptor-based analysis complements, rather than replaces, the image-based interpretation methods. Grad-CAM identifies image regions associated with the MDLF prediction, whereas the Random Forest-SHAP analysis relates the overall variation in the MDLF outputs to measurable microstructural descriptors.

13. **Transferability**

Although the present framework was developed and validated using the IN718 dataset, its overall architecture is not restricted to a single alloy system. The image encoder is initialized from ImageNet pretrained weights, whose convolutional layers mainly extract generic visual features such as edges, boundaries, textures and morphological patterns. These low-level representations are expected to remain applicable to SEM micrographs of other precipitation-strengthened Ni-based superalloys.

However, the higher-level feature representations learned by the multimodal framework are determined by alloy composition, heat-treatment history and the associated microstructural evolution. Different precipitation-strengthened Ni-based superalloys often exhibit distinct precipitation kinetics, phase constitutions and strengthening mechanisms, leading to different relationships between processing parameters, microstructure and creep rupture behavior. Consequently, directly applying the pretrained model to another alloy system may not provide optimal predictive performance.

For practical applications, a lightweight fine-tuning strategy using a relatively small alloy-specific dataset is expected to be more appropriate than training a completely new model from scratch. Such a strategy can preserve the generic feature extraction capability learned by the pretrained encoder while adapting the high-level multimodal representations to alloy-specific microstructural characteristics. Future work will investigate the transferability of the proposed framework across different precipitation-strengthened Ni-based superalloys through systematic transfer-learning studies.

14. **Construction of ML model**

**14.1 Data collection**

This study collected a dataset containing 266 sets of data from patent literature (Table S2), which was constructed to screen for process parameters that affect the creep rupture life (CRL) of IN718 alloy. Subsequently, Pearson correlation analysis was performed on the data, and the results are shown in Figure S13a-b: Solution treatment temperature (ST) and holding time (STt) are the two features with the strongest correlation with CRL. Therefore, the experimental process focuses on designing changes in these two parameters.

Modulating ST and STt induces microstructural variability. Such variation is crucial for accurate model construction. Three IN718 alloys with distinct compositions were fabricated into rods using melting, forging, rolling and cold drawing processes (Table S3-5). Application of heat treatments featuring stepwise variations in ST and STt (Figure S13c) served to modulate the volume fraction and dimensions of the δ-phase. Specifically, experiments were conducted at 980 ^o^C, proximate to the δ-phase dissolution temperature, to investigate the temporal effects of treatment. This specific temperature regime was selected to enable the selective dissolution of the intracrystalline δ-phase while retaining the grain boundary δ-phase to effectively pin grain boundaries and suppress grain growth.

**Figure S13.** Correlation analysis of processing parameters and experimental design. a) Pearson correlation between heat treatment parameters and CRL. The correlation matrix and bar chart show the strongest positive correlation for the solution temperature (ST) and time (STt), with coefficients of 0.73 and 0.54. b) Violin plot of the distribution of the logarithm of CRL for different solution treatment temperature ranges. A non-monotonic relationship is observed, with the highest creep performance in the 400-800 ^o^C range. c) Schematic of the heat treatment profiles. The solution treatment temperatures vary (960 ^o^C, 970 ^o^C, 980 ^o^C) and are followed by a two-step aging process (720 ^o^C for 480 min and 620 ^o^C for 480 min).

The heat-treated samples were mechanically ground and polished. Etching was performed for 3 s in a solution of 10 vol.% hydrochloric acid, 5 vol.% methanol and 3 g copper chloride. Microstructures were imaged using an AXIO Observer A1m optical microscope (OM) and a Zeiss ULTRA 55 scanning electron microscope (SEM). Stress rupture tests were conducted at 650 ^o^C and 690 MPa on an RMT-D10 electronic testing machine. The resulting 134 data points include processing parameters, composition, microstructural micrographs and creep rupture life.

**12.2 Data processing and model evaluation**

The 134 experimental samples were divided into training (94 samples), validation (20 samples) and test (20 samples) sets. Stratified random sampling was used to ensure a balanced distribution of alloy compositions across the subsets. Strict sample-level partitioning was applied to prevent data leakage. All data derived from a specific sample, including tabular features and image tiles, were assigned to a single subset. Numerical features, including alloy composition and heat treatment parameters (Table S4), were processed using min-max normalization. Application of a logarithmic transformation to the target variable, creep rupture life, (Eq. S14) functions to rectify distributional skewness and ensure variance stabilization.

$Y^{*}=\log\left( Y \right)$	(S14)

Data preparation followed established protocols for deep learning inputs.^[3-6]^ Original SEM micrographs measured 1920×1275 pixels. Six tiles of 224×224 pixels were cropped from each image. To fit ResNet-18 input requirements and memory limits, the micrographs were resized and converted to 3-channel format by duplicating the grayscale channel RGB format. The pixel intensity was then normalized using an ImageNet prior (mean: [0.485, 0.456, 0.406]; standard deviation: [0.229, 0.224, 0.225]). This step ensures the stability of the model in the convergence process, and expands the data set size through the data enhancement strategy. Horizontal flipping and random rotations (±15^o^) were applied without affecting microstructural morphology. The final dataset consists of 1596 samples. These were split into training (1128), validation (240), and testing (228) sets.

Assessment of deep learning accuracy utilized the metrics of *R^2^*, *RMSE* and *MAPE*, as formulated in equations (2), (3) and (4) respectively.^[7]^

$R^{2}=1-\frac{\sum_{i=1}^{n} \left( y_{measured}-y_{predicted} \right)}{\sum_{i=1}^{n} \left( y_{measured}-y_{mean} \right)}$	(S15)

$RMSE=\sqrt{\frac{\sum_{i=1}^{n} \left( y_{predicted}-y_{measured} \right)}{n}}$	(S16)

$MAPE=\frac{100\%\sum_{i=1}^{n} \left| \left( \frac{y_{predicted}-y_{measured}}{y_{measured}} \right) \right|}{n}$	(S17)

where *y_measured_*, *y_predicted_* and *y_mean_* represent the experimental, predicted, and mean experimental values of creep rupture life, respectively. *n* is the number of data points. *R^2^* assesses the proportion of variance in the dependent variable that is predictable from the independent variables, while *RMSE* and *MAPE* measure the magnitude of prediction errors.

Model interpretation is required to validate physical plausibility and identify underlying mechanisms. Gradient-weighted Class Activation Mapping (Grad-CAM)^[8]^ was adapted for regression tasks to address the black-box nature of deep neural networks. This technique was applied to interpret the multimodal fusion model. The method generates a visual "heat map" by first computing the gradients of the predicted creep rupture life with respect to the feature maps of the final convolutional layer in the ResNet-18 backbone. These gradients are then global-average-pooled to obtain importance weights, which are used to create a weighted combination of the feature maps. The resulting activation maps were upsampled using bilinear interpolation to match the original image dimensions and aggregated across multiple image patches to provide a comprehensive visualization. When overlaid on the input SEM image, this heat map provides a direct and intuitive visualization of the microstructural regions such as the morphology, size and distribution of the δ-phase that were most influential in the decision-making process of the model, thereby offering a powerful tool for mechanism discovery.

**Table S2.** Database of creep rupture life of Ni-based superalloys

| **Type** | **Variable** | **Symbol** | **Minimum** | **Maximum** |
| --- | --- | --- | --- | --- |
| Inputs | Ni (wt.%) | Ni | 46.707 | 75.715 |
|  | Chromium (wt.%) | Cr | 12.690 | 33.810 |
|  | Cobalt (wt.%) | Co | 0.000 | 25.000 |
|  | Iron (wt.%) | Fe | 0.000 | 20.477 |
|  | Aluminum (wt.%) | Al | 0.400 | 3.700 |
|  | Titanium (wt.%) | Ti | 0.047 | 5.040 |
|  | Niobium (wt.%) | Nb | 0.000 | 5.500 |
|  | Molybdenum (wt.%) | Mo | 0.000 | 9.830 |
|  | Tungsten (wt.%) | W | 0.000 | 10.410 |
|  | Temperature (^o^C) | T | 19.00 | 1200.00 |
|  | Solid solution temperature (^o^C) | ST | 0.00 | 1140.00 |
|  | Solid solution time (h) | STt | 0.00 | 48.00 |
|  | Stabilizing aging temperature (^o^C) | STat | 0.00 | 1150.00 |
|  | Stabilization aging time (h) | Stat | 0.00 | 75.00 |
|  | Aging temperature (^o^C) | AT | 0.00 | 950.00 |
|  | Aging time (h) | At | 0.00 | 36.00 |
| Outputs | Creep rupture life (h) | CRL | 20.00 | 1834.00 |

**Table S3.** Chemical compositions of IN718 alloys (%, mass fraction)

| **Alloy** | **Ni** | **Cr** | **Co** | **Fe** | **Al** | **Ti** | **Nb** | **Mo** | **Mn** |
| --- | --- | --- | --- | --- | --- | --- | --- | --- | --- |
| #1- IN718 | 52.57 | 18.34 | 0.33 | 18.74 | 0.59 | 0.98 | 5.32 | 2.88 | 0.06 |
| #2- IN718 | 54.11 | 17.86 | 0.24 | 18.62 | 0.58 | 1.03 | 5.47 | 3.07 | 0.08 |
| #3- IN718 | 52.78 | 19.00 | 0.30 | 18.78 | 0.52 | 0.95 | 5.13 | 2.94 | 0.04 |

**Table S4.** Average grain size statistics of IN718 alloy under different heat treatment conditions

| **Heat treatment processes** | **Average grain size(um)** |
| --- | --- |
| 960 ^o^C 60 min | 6.33 |
| 970 ^o^C 60 min | 6.39 |
| 980 ^o^C 60 min | 6.66 |
| 990 ^o^C 60 min | 7.42 |
| 1000 ^o^C 60 min | 32.00 |
| 1020 ^o^C 60 min | 71.09 |
| 980 ^o^C 0 min | 6.90 |
| 980 ^o^C 10 min | 6.52 |
| 980 ^o^C 20 min | 6.54 |
| 980 ^o^C 30 min | 6.60 |
| 980 ^o^C 120 min | 6.71 |
| 980 ^o^C 180 min | 6.90 |
| 970 ^o^C 90 min+970 ^o^C 90 min | 6.99 |
| 980 ^o^C 60 min+980 ^o^C 60 min | 7.21 |
| 970 ^o^C 60 min+980 ^o^C 60 min | 6.54 |

**Table S5.** Database of creep rupture life of Ni-based superalloys

| **Type** | **Variable** | **Symbol** | **Minimum** | **Maximum** |
| --- | --- | --- | --- | --- |
| Inputs | Ni (wt.%) | Ni | 46.707 | 75.715 |
|  | Chromium (wt.%) | Cr | 12.690 | 33.810 |
|  | Cobalt (wt.%) | Co | 0.000 | 25.000 |
|  | Iron (wt.%) | Fe | 0.000 | 20.477 |
|  | Aluminum (wt.%) | Al | 0.400 | 3.700 |
|  | Titanium (wt.%) | Ti | 0.047 | 5.040 |
|  | Niobium (wt.%) | Nb | 0.000 | 5.500 |
|  | Molybdenum (wt.%) | Mo | 0.000 | 9.830 |
|  | Tungsten (wt.%) | W | 0.000 | 10.410 |
|  | Temperature (^o^C) | T | 19.00 | 1200.00 |
|  | Solid solution temperature (^o^C) | ST | 0.00 | 1140.00 |
|  | Solid solution time (h) | STt | 0.00 | 48.00 |
|  | Stabilizing aging temperature (^o^C) | STat | 0.00 | 1150.00 |
|  | Stabilization aging time (h) | Stat | 0.00 | 75.00 |
|  | Aging temperature (^o^C) | AT | 0.00 | 950.00 |
|  | Aging time (h) | At | 0.00 | 36.00 |
| Outputs | Creep rupture life (h) | CRL | 20.00 | 1834.00 |

**References**

[1] L. Tan, X. G. Yang, D. Q. Shi, W. Q. Hao, and Y. S. Fan, “Unified fatigue life modelling and uncertainty estimation of Ni-based superalloy family with a supervised machine learning approach,” Engineering Fracture Mechanics 275 (2022): 108813. <https://doi.org/10.1016/j.engfracmech.2022.108813>.

[2] N. Hollmann, S. Müller, L. Purucker, et al., “Accurate predictions on small data with a tabular foundation model,” Nature 637 (2025): 319-326. <https://doi.org/10.1038/s41586-024-08328-6>.

[3] Y. Nie, A. S. Zamzam, and A. Brandt, “Resampling and data augmentation for short-term PV output prediction based on an imbalanced sky images dataset using convolutional neural networks,” Solar Energy 224 (2021): 341-354. <https://doi.org/10.1016/j.solener.2021.06.014>.

[4] X. Zhang, Z. Wang, D. Liu, Q. Lin, and Q. Ling, “Deep adversarial data augmentation for extremely low data regimes,” IEEE Transactions on Circuits and Systems for Video Technology 31 (2021): 15-28. <https://doi.org/10.1109/TCSVT.2020.2970564>.

[5] S. M. Azimi, D. Britz, M. Engstler, M. Fritz, and F. Mücklich, “Advanced steel microstructural classification by deep learning methods,” Scientific Reports 8 (2018): 2128. <https://doi.org/10.1038/s41598-018-20037-5>.

[6] B. Ma, X. Ban, H. Huang, Y. Chen, W. Liu, and Y. Zhi, “Deep learning-based image segmentation for Al-La alloy Microscopic Images,” Symmetry 10 (2018): 107. <https://doi.org/10.3390/sym10040107>.

[7] H. Anysz, A. Zbiciak, and N. Ibadov, “The Influence of Input Data Standardization Method on Prediction Accuracy of Artificial Neural Networks,” Procedia Engineering 153 (2016): 66-70. <https://doi.org/10.1016/j.proeng.2016.08.081>.

[8] F. Chollet 2018, Deep Learning with Python. New York: Manning Publications.

1. ^*^Corresponding author:

   E-mail: wudayong_ysu@126.com; ziyuanrao@sjtu.edu.cn; zny@ncst.edu.cn; sxru2008@163.com.
